# MKL1 cooperates with p38MAPK to promote vascular senescence, inflammation, and abdominal aortic aneurysm

**DOI:** 10.1016/j.redox.2021.101903

**Published:** 2021-02-20

**Authors:** Ping Gao, Pan Gao, Jinjing Zhao, Shengshuai Shan, Wei Luo, Orazio J. Slivano, Wei Zhang, Akiko Tabuchi, Scott A. LeMaire, Lars Maegdefessel, Ying H. Shen, Joseph M. Miano, Harold A. Singer, Xiaochun Long

**Affiliations:** a Department of Molecular and Cellular Physiology, Albany Medical College, Albany, NY, USA; b Vascular Biology Center, Medical College of Georgia at Augusta University, Augusta, GA, USA; c Michael E. DeBakey Department of Surgery, Baylor College of Medicine, One Baylor Plaza, BCM 390, Houston, TX, USA; d Laboratory of Molecular Neurobiology, Graduate School of Medicine & Pharmaceutical Sciences, University of Toyama, 2630 Sugitani, Toyama, 930-0194, Japan; e Department for Vascular and Endovascular Surgery, Klinikum Rechts der Isar, Technical University Munich, Germany; f German Center for Cardiovascular Research (DZHK, Partner Site Munich), Germany; g Department of Medicine, Karolinska Institute, Stockholm, Sweden

**Keywords:** Aortic aneurysm, MKL1, p38MAPK, Senescence, Inflammation

## Abstract

Abdominal aortic aneurysm (AAA) is a catastrophic disease with little effective therapy. Myocardin related transcription factor A (MRTFA, MKL1) is a multifaceted transcription factor, regulating diverse biological processes. However, a detailed understanding of the mechanistic role of MKL1 in AAA has yet to be elucidated. In this study, we showed induced MKL1 expression in thoracic and abdominal aneurysmal tissues, respectively in both mice and humans. MKL1 global knockout mice displayed reduced AAA formation and aortic rupture compared with wild-type mice. Both gene deletion and pharmacological inhibition of MKL1 markedly protected mice from aortic dissection, an early event in Angiotensin II (Ang II)-induced AAA formation. Loss of MKL1 was accompanied by reduced senescence/proinflammation in the vessel wall and cultured vascular smooth muscle cells (VSMCs). Mechanistically, a deficiency in MKL1 abolished AAA-induced p38 mitogen activated protein kinase (p38MAPK) activity. Similar to MKL1, loss of MAPK14 (p38α), the dominant isoform of p38MAPK family in VSMCs suppressed Ang II-induced AAA formation, vascular inflammation, and senescence marker expression. These results reveal a molecular pathway of AAA formation involving MKL1/p38MAPK stimulation and a VSMC senescent/proinflammatory phenotype. These data support targeting MKL1/p38MAPK pathway as a potential effective treatment for AAA.

## Highlights

• MKL1 expression is induced in both thoracic and abdominal aneurysmal tissues.
• Genetic ablation and pharmacological inhibition of MKL1 protect mice from aortic dissection and AAA induced by Ang II.
• Depletion of MKL1 in mice suppresses Ang II-induced vascular inflammation and senescence.
• Depletion of MKL1 blunts the activation of p38MAPK and STAT3 pathways.
• Loss of MAPK14 in VSMCs suppresses Ang II-induced AAA formation, vascular inflammation, and senescence marker expression.

## Graphical abstract

**Figure:**
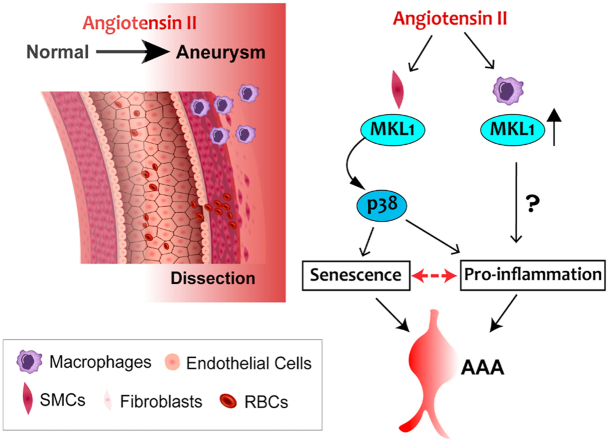

## 1 Introduction

Aortic aneurysm is permanent dilatation, largely occurring in thoracic and abdominal segments of aortas, thus referred to as thoracic aortic aneurysm (TAA) and abdominal aortic aneurysm (AAA), respectively. Though genetic distinctions, such as population prevalence, inheritance means, and risk genes exist in the etiology of TAA and AAA, each shares certain pathological similarities, including elastic tissue degeneration, smooth muscle cell (SMC) loss, and rupture [1]. AAA is the most common aortic aneurysm, defined by weakening and localized dilatation of the abdominal aorta (AA) with a diameter exceeding 50% greater than the normal diameter [[2], [3], [4]]. AAA grows asymptomatically until rupture occurs, which is catastrophic and carries a mortality of 65%–85% of cases [5]. Beyond risky open surgical or endovascular intervention for large AAA, there is currently no proven pharmaceutical treatments to prevent AAA progression. The pathophysiology of AAA is highly complex, which is underpinned by an interplay of genetic risk factors, such as a positive family history and male sex, and environmental exposures, including cigarette smoking, hypertension, and coronary artery disease [5,6]. AAA is a degenerative vascular complication, pathologically hallmarking vascular smooth muscle cell (VSMCs) depletion, dysregulation of reactive oxygen species (ROS), extracellular matrix (ECM) degradation, and inflammatory cell infiltration [6]. An intense inflammatory response in the vessel wall has long been recognized as a crucial trigger for AAA formation [6,7]. In addition, recent studies have linked cellular senescence to AAA pathogenesis [8,9]. Therefore, unveiling key regulator(s) that dictate vascular inflammation and senescence is essential for developing effective therapies to AAA.

Myocardin related transcription factors (MRTFs), including Myocardin (MYOCD), Myocardin-related transcription factor A (MRTFA, MKL1), and Myocardin-related transcription factor B (MRTFB, MKL2), comprise a family of coactivators that potentiate the transcriptional activity of serum response factor (SRF) [10,11]. In contrast to MYOCD, a component of a molecular switch of VSMC differentiation [12], the precise function of MRTFs in the adult cardiovascular system remains incomplete. Though a similar role to MYOCD in promoting vascular smooth muscle differentiation has been reported [13,14], MKL1 is unable to compensate for loss of MYOCD in the transactivation of VSMC contractile gene program [15]. Further, global deficiency of MKL1 fails to exert an adult vascular phenotype under physiological conditions [14]. Therefore, MKL1 is seemingly dispensable for vascular homeostasis. In contrast, a human genetic screening study revealed that enhanced MKL1 activity was associated with susceptibility to coronary artery diseases [16]. Increased MKL1 activity underlies several vascular complications, including restenosis, atherosclerosis, and vascular fibrosis [17,18]. This is likely attributable to disparate gene programs activated by MKL1 to necessitate vascular maladaptation, such as VSMC migration, macrophage cell apoptosis, excessive activation of ROS, and vascular stiffness. However, a detailed understanding of the mechanistic role of MKL1 in AAA development and progression has yet to be elucidated.

The stress responsive pathway, p38MAPK, has been reported as a pivotal regulator in inflammation, proliferation, migration, and apoptosis, and contributes to a number of vascular diseases [19,20]. However, the definitive role of MAPK14, which encodes p38α, the major isoform of the p38MAPK family in VSMCs, and its upstream regulator(s) during AAA formation, remain to be explored.

In the present study, we show that MKL1 expression was induced in TAA and AAA tissues from both mice and humans. Through genetic and pharmacological approaches, we demonstrate a critical pro-aneurysmal role for MKL1 in a well-recognized angiotensin II (Ang II)-induced AAA mouse model. We provide evidence that supports MKL1 in promoting vascular senescence and inflammation, likely through a VSMC-p38MAPK pathway. These findings may provide important insight into novel therapeutic strategies for AAA via targeting MKL1 and the p38MAPK pathway.

## 2 Material and methods

### 2.1 Mouse experiments

All animal experiments were conducted in accordance with experimental protocols that were approved by the Institutional Animal Care and Use Committee (IACUC) at Albany Medical College. All mice were of C57BL/6J background and born at the expected Mendelian ratio. *Mkl1* global knockout (*Mkl1*^*−/−*^) mice [14] were bred with ApoE (apolipoprotein E) knockout (*Apoe*^*−/−*^) mice (Jackson Laboratory) to generate *Mkl1*^*−/−*^, *Apoe*^*−/−*^ (KO) mice. Since female *Mkl1*^*−/−*^mice are unable to nurse their offspring due to dysfunction of the mammary gland [14], female *Mkl1*^+/−^, *Apoe* *^−/−^* mice were utilized to breed with male *Mkl1*^+/−^, *Apoe*^*−/−*^ or *Mkl1*^−/−^, *Apoe*^*−/−*^ mice for generating KO. To minimize animal breeding, *Mkl1*^+/+^, *Apoe*^*−/−*^ (WT) mice from either littermate or sibling mate were used as control to KO mice. *Sm22-Cre* and *Mapk14*^f/f^ mice were obtained as described previously [21,22] and bred to generate *Sm22-Cre*, *Mapk*14^f/f^ mice for SMC-specific deletion of *Mapk14*. *Sm22*-*Cre,* *Mapk14*^f/f^ mice were then bred to *Apoe*^*−/−*^ to generate *Sm22-Cre,* *Mapk14*^f/f^, *Apoe*^*−/−*^ mice. Both *Mapk14* WT control mice from littermate (*Mapk14*^f/f^, *Apoe*^*−/−*^) and sibling mate (*Sm22-Cre,* *Mapk14*^+/+^, *Apoe*^*−/−*^) were utilized. 6 month-old male mice were used for experiments infused with Ang II (A9525, Sigma-Aldrich) for 7 or 28 days subcutaneously (1 mg/kg/min, 1.44 mg/kg/day) by implantation of Alzet osmotic minipumps (Model 1004, Alzet, Cupertino, CA) per the published protocol [23]. Given the lower incidence of Ang II- induced AAA model in females and the protective phenotype of both *Mkl1*^*−/−*^, *Apoe*^*−/−*^ and *Sm22*-*Cre*, *Mapk14*^f/f^, *Apoe*^*−/−*^ mice, we limited our experiments to male mice. For MKL1 inhibitor experiments, CCG-1423 (Cayman Chemical Company, MI) was dissolved in 30% DMSO/15% ethanol/55% PBS and administrated at a dose of 1 mg/kg/day via intraperitoneal injection from 3 days prior to until 7 days post Ang II pump implantation.

The mice that died after Ang II infusion were immediately necropsied to determine the cause of death. For sample collection, mice were euthanized with pentobarbital (150 mg/kg, i. p), aortas were perfused with saline, dissected, and cleaned by removing the surrounding connective tissues. AAA was defined as a 50% or greater increase in the external width of the AA compared to that from saline-infused mice. AAA incidence was calculated as the percentage of AAA formation to total mouse number per group. Measurements of maximal diameter and Doppler ultrasound imaging of AA luminal diameter were conducted as previously described [24,25].

### 2.2 Human sample study

Human abdominal aortic tissue specimens were provided by the Munich Vascular Biobank from patients undergoing elective open AAA repair and organ donors (kidney transplant surgery) [26]. Human ascending thoracic aortic aneurysm and dissection and control normal aortic segments were from our existing aortic tissue bank as described in our recent publication [27], respectively. The related protocols were approved by the Institutional Review Board (IRB) at the Klinikum rechts der Isar of the Technical University Munich and Baylor College of Medicine, respectively. The experiments were conducted in accordance with the guidelines and regulations described in the individual IRB protocols. We utilized portion of AAA segments for qRT-PCR and TAA for Western blot of MKL1 per our standard methods descried below. Patients’ characteristics are included in Supplementary Tables 1 and 2

### 2.3 Immunofluorescence staining

Portions of suprarenal or thoracic aortas were collected and fixed with 4% paraformaldehyde overnight at 4 °C before paraffin embedding for sectioning. Cross sections (5 μm) were collected serially at intervals of 100 μm for immunostaining as described previously [20]. Sections were deparaffinized, rehydrated, followed by high pressure-mediated antigen retrieval for 10 min. Sections were permeabilized, blocked with a blocking solution (Dako, X0909) for 30 min at room temperature (RT), and probed for the indicated primary antibody overnight at 4 °C and second antibody for 1 h at RT. Finally, slides were mounted with Fluoroshield™ with DAPI (F6057, Sigma-Aldrich) before image acquisition. Fluorescence images were captured by confocal microscopy (Leica DMI4000 B, Leica Microsystems) and the relative signal intensity was quantified by Image J. Identical acquisition parameters were utilized for each experiment. Information on primary and secondary antibodies is described in Supplementary Table 3.

### 2.4 Histological analyses

After deparaffinization and rehydration, mouse sections were subjected to standard hematoxylin and eosin (H&E), Masson trichrome, or Verhoeff-van Gieson (VVG) (HT25A, Sigma-Aldrich) staining for morphometric analyses. Elastin degradation score was analyzed as described [28]. Collagen content was quantified as the fraction of positive staining area within the whole area using Image J.

### 2.5 Senescence-associated β-galactosidase (SA-β-gal) staining

SA-β-gal staining was conducted to measure senescence using a commercial kit (ab65351, Abcam) according to the manufacturer's instruction. Briefly, freshly isolated aortas were fixed in 4% paraformaldehyde overnight at 4 °C and washed with PBS followed by the incubation with the staining solution at 37 °C for 24 h. Cells with blue color were considered SA-β-gal positive and senescent cells. SA-β-gal positive area was quantified by Image J. Serial cross sections (5 μm) from SA-β-gal stained aortas were collected for imaging to further assess the distribution of senescent cells.

### 2.6 Cell culture and treatment

Primary human and mouse aortic SMCs (HASMCs and MASMCs) were prepared and maintained as previously described [20]. Bone marrow derived macrophages (BMDMs) were prepared according to a published protocol [24]. SK-LMS-1 (SKLMS) were obtained from ATCC and maintained in DMEM medium (Gibco) supplemented with 10% fetal bovine serum. Generation and transduction of lentivirus carrying short hairpin RNA targeting *MKL1* (lenti-shMKL1) and control vector (lenti-shCtrl) were conducted as described previously [29]. HASMCs were fed growth medium containing equal amount of shMKL1 and shCtrl supplemented with polybrene (TR-1003, Sigma-Aldrich) for 24 h. Cells were then refreshed with growth medium for another 48 h followed by Ang II treatment (100 nM, Sigma-Aldrich) for 24 h for RNA isolation or 48 h for protein isolation. For IL1β (5 ng/ml, 201-LB-005, R&D Systems) and TNFα (10 ng/ml, 210-TA-005, R&D Systems) induction, cells were serum-starved overnight prior to the stimulation for 0.5–1 h as indicated in the individual figure legend. The transduction of VSMCs with adenovirus carrying the constitutively active MKK6 (Ad-MKK6) and the negative control empty adenovirus (Ad-empty) were described previously [30].

### 2.7 RNA isolation and quantitative reverse Transcriptase-PCR (qRT-PCR)

Periadventitial adipose tissue-free mouse aortas and human aortic tissues were homogenized by a Minilys homogenizer (Bertin Technologies, Rockville, MD) using Precellys Lysing Kit (VWR Scientific, Radnor, PA). Total RNA from homogenized aortic tissues or cultured cells was extracted using miRNeasy mini kit (217004, Qiagen). The concentration of RNA was measured using a Nanodrop 2000 spectrophotometer (Thermo Fisher Scientific). 200–500 ng (ng) of total RNA was used for cDNA synthesis using iScript cDNA kit (Bio-Rad). qRT-PCR was carried out using Universal SYBR Green Supermix (Bio-Rad) and CFX386 Touch™Real-Time PCR Detection System (Bio-Rad). Sequences of primers used in this study are listed in Supplementary Table 4.

### 2.8 Western blot analysis

Freshly isolated and periadventitial adipose tissue-free aortas and human TAA tissues were homogenized by a Minilys homogenizer (Bertin Technologies) using Precellys Lysing Kit (VWR Scientific). Total protein from homogenized aortic tissues or cultured cells was extracted with ice-cold lysis buffer (9803, Cell Signaling Technology) supplemented with a protease inhibitor cocktail (Sigma). Protein was resolved on SDS-PAGE gel to probe for with the indicated antibodies as described previously [20]. Information of antibodies for Western blot is provided in Supplementary Table 3.

### 2.9 RNA sequencing (RNA-seq)

Total RNA of cultured BMDMs from WT and KO mice with Ang II Infusion for 7 days was subjected to bulk RNA-seq conducted by Genomics Research Center at the University of Rochester Medical Center. Detailed information of library construction and data analysis was described previously [31]. Heatmap was illustrated using the raw count value of all related transcripts and presented as the log transformation of the normalized raw count data. *Mkl1* gene expression in different cell clusters in control versus aneurysmal aortas induced by Ang II infusion combined with high fat diet, was based on our recently published single cell RNA-seq study [27]. We used dimensional reduction and shared nearest neighbor (SNN) modularity clustering algorithm to assess cell population deconvolution. Cell clusters were defined according to a set of highly expressed differential/conservative/cell marker genes. High expression levels of SMC marker genes (*Acta2*, *Myh11*, *Mylk*), macrophage marker genes (*Cd68*, *Adger1*, *F13a1*), and endothelial cell (EC) marker genes (*Pecam1*, *Vwf*) were utilized to define each cell type. Within each cluster, *Mkl1* was identified as one of differentially expressed genes (DEGs) between two groups of cells by using a Wilcoxon rank sum test.

### 2.10 Statistical analyses

All experiments were repeated independently at least three times. Statistical analyses were performed using GraphPad Prism 7.0. Quantitative results were presented as mean ± standard error of mean (SEM). The normality and equal variance of the data were assessed. For comparison between 2 groups, unpaired 2-tailed Student t-test was used to determine statistical difference for normally distributed variables, and nonparametric Mann-Whitney test was used for data without normal distribution. Welch's correction was applied for data with unequal variance. For comparison of multiple groups, two-way ANOVA followed by Sidak multiple comparison test was used for normally distributed variables. Fisher's exact test and log-rank test were used for analyzing AAA incidence and survival, respectively. Repeated-measure two-way ANOVA was used for blood pressure experiment. p < 0.05 was considered statistically significant.

## 3 Results

### 3.1 MKL1 expression is induced in both AAA and TAA lesions in mice and humans

**Fig. 1:**
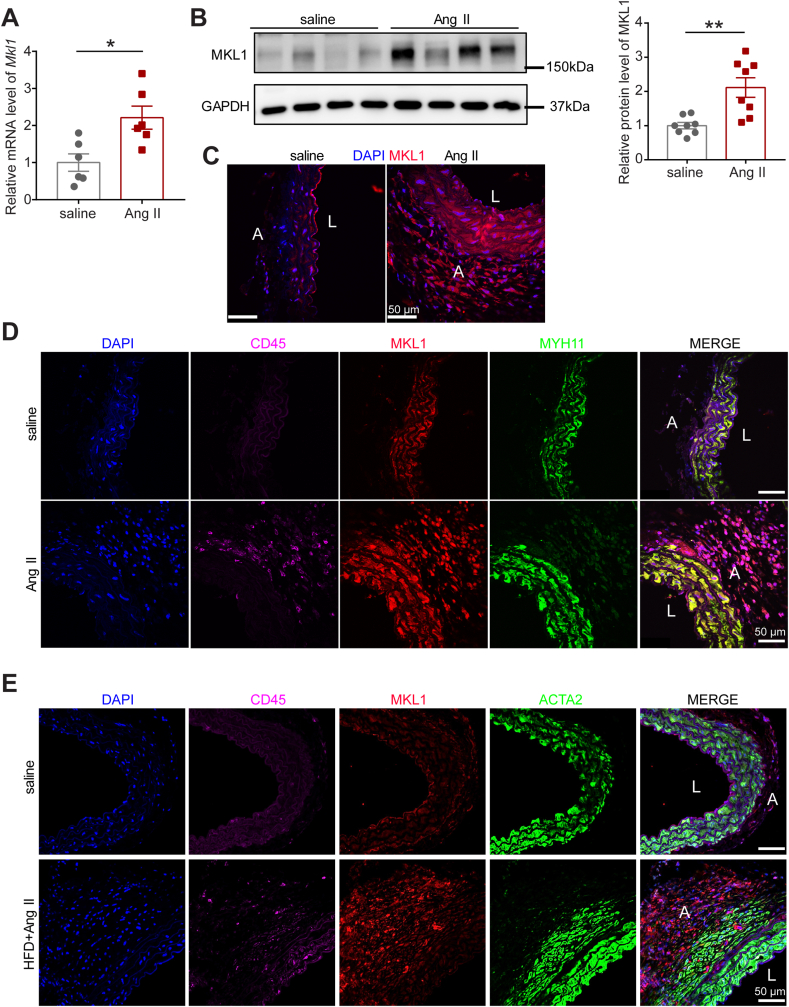

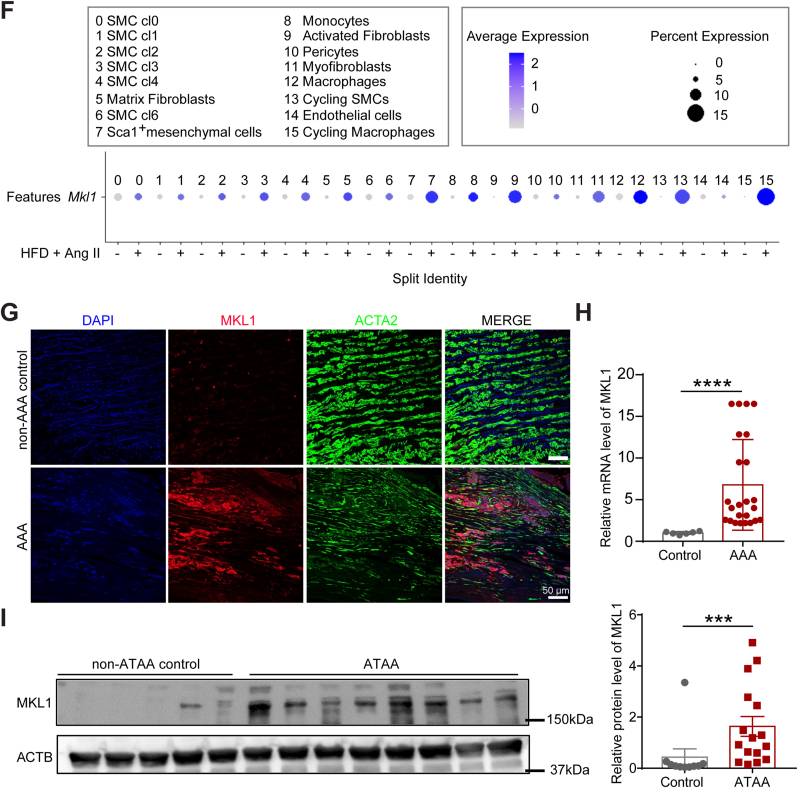
MKL1 expression is induced in both AAA and TAA lesions in mice and humans. A, qRT-PCR analysis of *Mkl1* in total RNA extracted from aortas of *Apoe−/−* mice exposed to saline or angiotensin II (Ang II) for 1 week (n = 6). Unpaired two-tailed Student's *t*-test. B, Representative Western blot images and quantification of MKL1 in aortas of *Apoe−/−* mice exposed to saline or Ang II for 1 week (n = 8). Unpaired two-tailed Student's *t*-test with Welch's correction. C and D, Representative confocal microscopy images for immunofluorescence staining of the indicated proteins in cross-sections of the suprarenal aortas from *Apoe−/−* mice infused with Ang II or saline for 1 week (n = 3). E, Representative confocal microscopy images for immunofluorescence staining of the indicated proteins in cross-sections of the thoracic aortas from C57BL/6 mice infused with high fat diet (HFD) with Ang II or saline for 4 weeks (n = 3). F, Dot plot graph from single-cell RNA-sequencing analysis [27] in aortas of C57BL/6 mice fed with HFD plus Ang II infusion vs C57BL/6 mice fed with chow diet without Ang II infusion (n = 3, pooled). The size of circle represents the percent of cells expressing *Mkl1* in each cluster; the color scale indicates the expression level. G, Representative confocal microscopy images for immunofluorescence staining of the indicated proteins in cross-sections of human AAA versus non-aneurysmal aortic tissues (n = 3). H, qRT-PCR analysis of *MKL1* in total RNA extracted from control aortic tissues (control, n = 6) from organ donors versus human AAA samples (AAA, n = 24). Mann-Whitney test. I, Western blot image and the quantitation for MKL1 protein in aortic tissues from organ donors (control, n = 10) and patients with ascending thoracic aortic aneurysm (ATAA, n = 16). Mann-Whitney test. *P < 0.05, **P < 0.01, ***P < 0.001. (For interpretation of the references to color in this figure legend, the reader is referred to the Web version of this article.)

To determine if MKL1 is associated with aortic aneurysm development, we assessed its expression in aortas from *Apoe*^*−/−*^ mice subjected to Ang II infusion, a well-recognized experimental aortic aneurysm model. Aortas infused with Ang II for 1 week normally exhibit typical pathological manifestations of aneurysm, including aortic dilation, media interruption, macrophage infiltration, and VSMC apoptosis [32,33] and therefore were assayed for MKL1 expression. qRT-PCR showed that *Mkl1* mRNA was elevated in aortas infused with Ang II relative to saline controls (Fig. 1A). Similar induction of MKL1 protein was confirmed by Western blot (Fig. 1B). Immunofluorescence staining revealed an upregulation of MKL1 protein in both medial layer and adventitia of suprarenal aortas triggered by Ang II (Fig. 1C). To identify cell-specific induction of MKL1 protein, we carried out double staining for MKL1 with either MYH11, a definitive marker protein for SMC lineage, or CD45, a leucocyte marker in suprarenal aortas from saline or Ang II-infused *Apoe*^*−/−*^ mice. Co-localization of MKL1 and MYH11 was observed in suprarenal aortas from both groups, indicative of induction of MKL1 in VSMCs. There was a large number of CD45 positive cells with strong MKL1 staining accumulated in the adventitia of Ang II-infused suprarenal aortas (Fig. 1D). Similar induction was seen in TAA tissues compared with normal thoracic aortas (TAs) in a high fat diet and Ang II infusion aortic aneurysm mouse model (Fig. 1E) [34]. Consistently, single cell RNA-seq (scRNA-seq) analysis of TAA lesion versus control TAs in this model showed increased expression of *Mkl1* transcript in 7 different SMC clusters and other cell types, such as activated fibroblasts, monocytes, pericytes, myofibroblasts, as well as macrophages (Fig. 1F). These results show that MKL1 was induced in SMCs as well as other vascular cell types during aortic aneurysm formation. To translate the above findings to humans, we firstly performed MKL1 immunostaining in human abdominal aneurysm tissues and non-aneurysm control aorta segments. MKL1 protein staining was much stronger in AAA lesions compared with control non-aneurysm aortic tissues, whereas ACTA2, a SMC marker protein, was reciprocally expressed in those tissues, indicating that VSMC phenotypic switching occurs during AAA formation in humans (Fig. 1G). Consistently, qRT-PCR showed more than 6-fold increase of *MKL1* mRNA expression in human AAA tissues relative to non-diseased aortas from organ donors (Fig. 1H). Finally, Western blot for human ascending thoracic aortic aneurysm (ATAA) tissues and normal aorta segments from organ donors revealed that MKL1 protein levels were substantially higher in ATAA samples (Fig. 1I). Taken together, these data demonstrate increased MKL1 expression in both AAA and TAA lesions in mice and humans.

### 3.2 Deletion of MKL1 protects mice from Ang II-induced AAA formation and aortic rupture

**Fig. 2:**
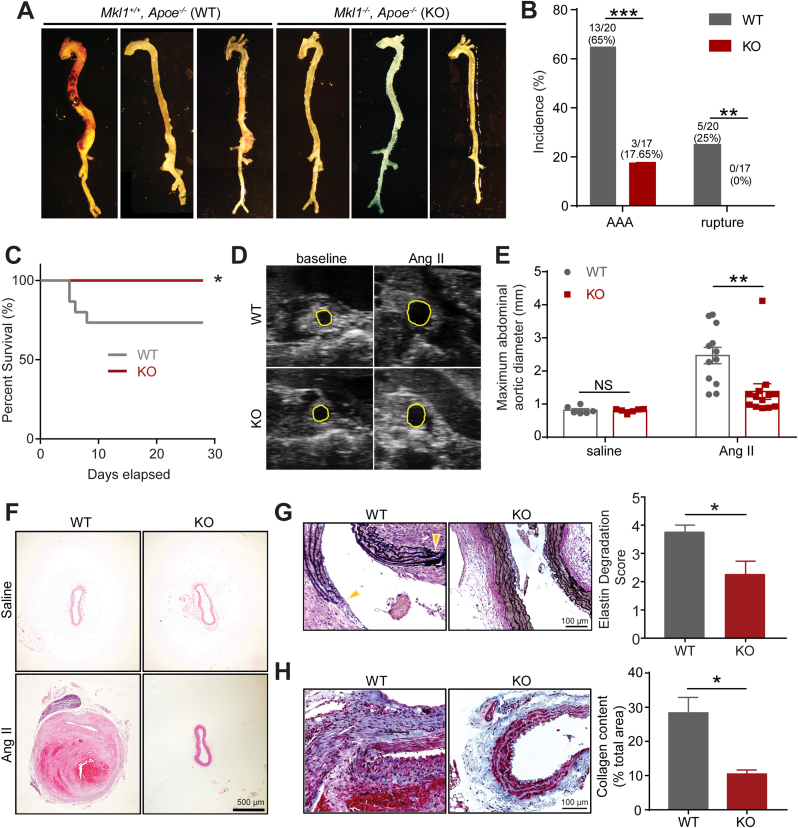
Deletion of MKL1 protects mice from Ang II-induced AAA formation and aortic rupture. All mice were infused with saline or Ang II for 4 weeks. A, Representative en face photographs showing aortas from *Mkl1*^*+/+*^*, Apoe*^*−/−*^ (WT) and *Mkl1*^*−/−*^*, Apoe*^*−/−*^ (KO) mice. B and C, The incidence of AAA and aortic rupture (B) and Kaplan-Meier survival curve (C) in Ang II-infused WT (n = 20) and KO (n = 17) mice. Chi-square test for (B), Log-rank test for (C). D, Representative Doppler ultrasound images of abdominal aortas before (baseline) and 4 weeks after Ang II infusion (n = 5). E, The maximal abdominal aortic diameter (the width of the maximal expanded portion of suprarenal aorta) quantified from excised aortas as shown in A (n = 6/group for saline controls; n = 12 for WT and n = 13 for KO were included for Ang II infused aortas). Two-way ANOVA followed by Sidak multiple comparison test. F–H, Representative images and quantification (unpaired two-tailed Student's *t*-test) of hematoxylin and eosin (H&E) (F), Verhoeff-Van Gieson (VVG) (G), and Masson trichrome staining (H) of cross-sections of suprarenal aorta from WT and KO mice (n = 4). Quantifications were performed on at least 3 separate sections from 4 animals from each group, the average value from all the sections of a given animal was used for comparison. Location of extensive elastin degradation was labeled with yellow arrowhead. *P < 0.05, **P < 0.01, ***P < 0.001, NS: not significant. (For interpretation of the references to color in this figure legend, the reader is referred to the Web version of this article.)

Given that AAAs are the most common aortic aneurysms occurring in humans, we sought to determine the functional role of MKL1 in AAA development. Age-matched (6 months) male *Mkl1*^*+/+*^, *Apoe*^*−/−*^ (WT) and *Mkl1*^*−/−*^, *Apoe*^*−/−*^ (KO) mice were challenged with Ang II infusion for 4 weeks. MKL1 deficiency was confirmed in aortas from KO mice by qRT-PCR, Western blot, and immunostaining (Supplementary Fig. 1A–C). KO mice developed normally and their systolic blood pressure was comparable to WT mice both prior to and 4 weeks post Ang II infusion (data not shown). There was no significant difference in blood cell count between the WT and KO mice after infusion with Ang II for 4 weeks (Supplementary Table 5). There was no aneurysm formation in both WT and KO mice infused with saline control for 4 weeks, and no distinguishable difference was seen in the gross morphology of aortas under this baseline condition (data not shown). 4 weeks of Ang II infusion led to a 66.7% incidence of AAA formation (13/20) in WT mice compared with only 17.65% (3/17) in KO mice (Fig. 2A and B). In our study, the majority of abdominal aortas (AAs) were characterized by prominent bulging containing transmural thrombus in the suprarenal region, which was categorized as Type III AAA according to the measurement protocol for this model (Fig. 2A) [35]. 5 out of 20 WT mice died due to aortic rupture within the first week of Ang II infusion. By contrast, all KO mice survived through the 4-week Ang II exposure (Fig. 2B and C). To monitor the dilation of the AA in vivo, we performed Doppler ultrasound imaging before and after 4 weeks of Ang II infusion. Ang II induced an evident dilation of suprarenal aortas in WT mice, which was sharply attenuated in KO mice (Fig. 2D). This result was confirmed by ex vivo maximal diameter measurement of AAs, which revealed a significantly enlarged external diameter of AAs in WT mice relative to KO after Ang II infusion for 4 weeks; saline-infused control mice from both groups showed comparable AA diameters (Fig. 2E). Histologically, 4 weeks of Ang II infusion in WT mice resulted in the formation of intraluminal thrombus, resulting from medial dissection evidenced by erythrocyte extravasation in H&E staining (Fig. 2F). Severe degradation of elastin lamina and matrix remodeling of the adventitia was also observed in the suprarenal aortas (Fig. 2G and H). These pathological structural damages largely occurred in Ang II-infused WT but not KO mice. Taken together, these results demonstrate a strong role for MKL1 in promoting AAA formation and aortic rupture.

### 3.3 Depletion of MKL1 inhibits Ang II-induced vascular cell senescence

**Fig. 3:**
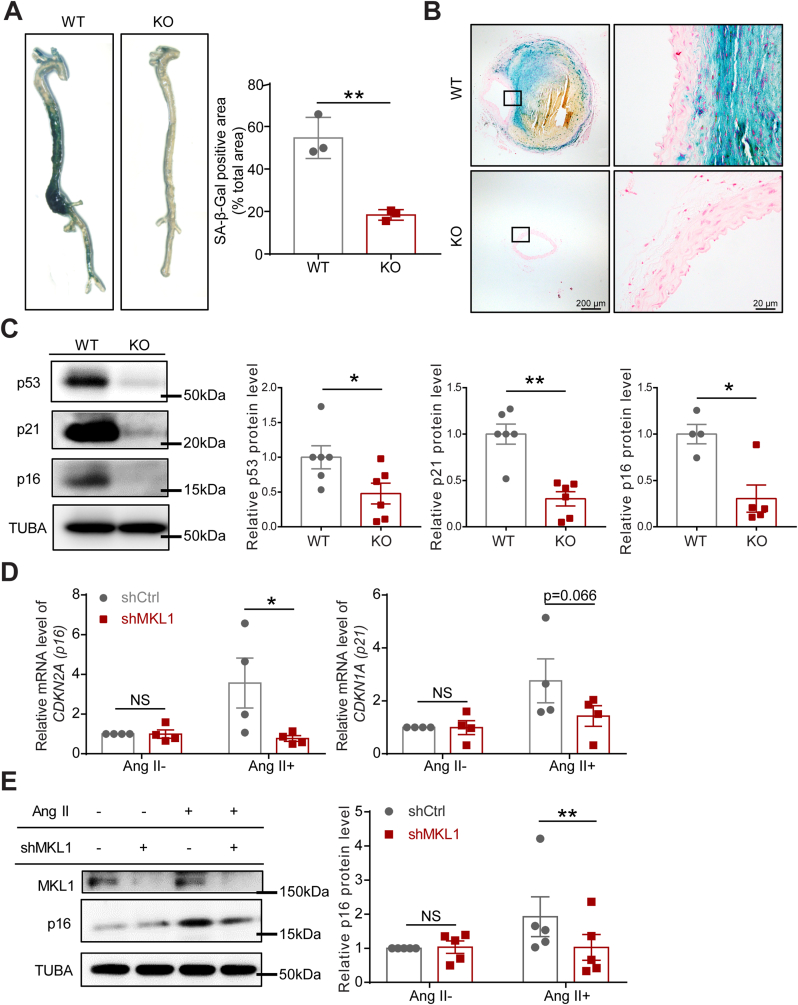
Depletion of MKL1 inhibits Ang II-induced vascular cell senescence. A and B, Representative images and quantification (unpaired two-tailed Student's *t*-test) of the senescence-associated beta-galactosidase (SA-β-gal)-stained whole aorta (n = 3) (A) and cross-sections of the suprarenal aorta from *Mkl1*^*+/+*^*, Apoe*^*−/−*^ (WT) and *Mkl1*^*−/−*^*, Apoe*^*−/−*^ (KO) mice infused with Ang II for 4 weeks (n = 5) (B). C, Representative Western blot images and quantification of senescence markers in aorta homogenates of WT and KO mice infused with Ang II for 1 week (n = 6 for p53 and p21, unpaired two-tailed Student's *t*-test; n = 4 for p16, Mann–Whitney test). D and E, qRT-PCR analysis (n = 4) (D) and representative Western blot images and quantification (n = 5) (E) of relative levels of MKL1 and senescence markers from human aortic smooth muscle cells (HASMCs) transduced with control or shMKL1 lentivirus for 72 h followed by Ang II (100 nM) or vehicle treatment. Pair matched two-way ANOVA followed by Sidak multiple comparison test. *P < 0.05, **P < 0.01, NS: not significant.

There is accumulating evidence supporting the contribution of cellular senescence to the pathogenesis of age-related vascular diseases such as atherosclerosis and aortic aneurysm [8,9,36,37]. Consistent with this notion, Western blot showed that one of the senescence markers, p21, was markedly induced in aortas from WT mice infused with Ang II (Supplementary Fig. 2A). We asked if the protective role of MKL1 deficiency in AAA development was attributable to its influence on senescence induced by Ang II. We first stained for SA-β-Gal activity, a well-recognized biomarker of cellular senescence in whole aortas from Ang II infused WT and KO mice. Positive SA-β-Gal staining was seen in the entire aorta while more robust signal was limited in AAs of WT mice infused with Ang II for 4 weeks. In contrast, there was barely detectable SA-β-gal staining in aortas of KO mice (Fig. 3A). A closer examination on suprarenal aortic sections showed that SA-β-Gal positive cells were predominantly located in the remodeled adventitia and its adjacent medial smooth muscle cell (SMC) layer (Fig. 3B). Western blot analysis of aortas cleaned of periadventitial tissue showed that several key senescence marker proteins, including p16, p53, and p21, were significantly reduced in aortas from KO compared with WT after 1 week Ang II infusion (Fig. 3C). Of note, saline-infused WT and KO mice displayed undetectable levels of p16 and insignificant difference in levels of p21 and p53 protein (Supplementary 2B). These results indicate that loss of MKL1 prevented vascular cell senescence in Ang II-induced AAA model.

It has been increasingly recognized that VSMC phenotypic modulation and reprograming play a critical role in aneurysm formation [38,39]. In addition, VSMC senescence has been reported to contribute to aneurysm formation [9]. To determine the specific role of VSMC-MKL1 in Ang II-triggered senescence, we depleted MKL1 in cultured primary HASMCs using lenti-shMKL1 followed by Ang II induction. Consistent with the in vivo data, MKL1 depletion inhibited the upregulation of the gold standard senescence marker, *CDKN2A* (p16) by Ang II, at both mRNA and protein levels; no such effect was seen at baseline (Fig. 3D, left panel, E). Similar results were observed in MASMCs isolated from *Mkl1*^*−/−*^ mice and *Mkl1*^*+/+*^ littermates (Supplementary Fig. 2C). In addition, the mRNA levels of *CDKN1A* (p21) showed an insignificant downregulation by lenti-shMKL1 in HASMCs induced by Ang II (Fig. 3D, right panel), and adenoviral-mediated MKL1 overexpression in HASMCs significantly induced p21 protein expression (Supplementary Fig. 2D). Collectively, these findings suggest that loss of MKL1 in VSMCs dampens vascular senescence under conditions of AngII-induced AAA formation.

### 3.4 Depletion of MKL1 suppresses vascular inflammation

**Fig. 4:**
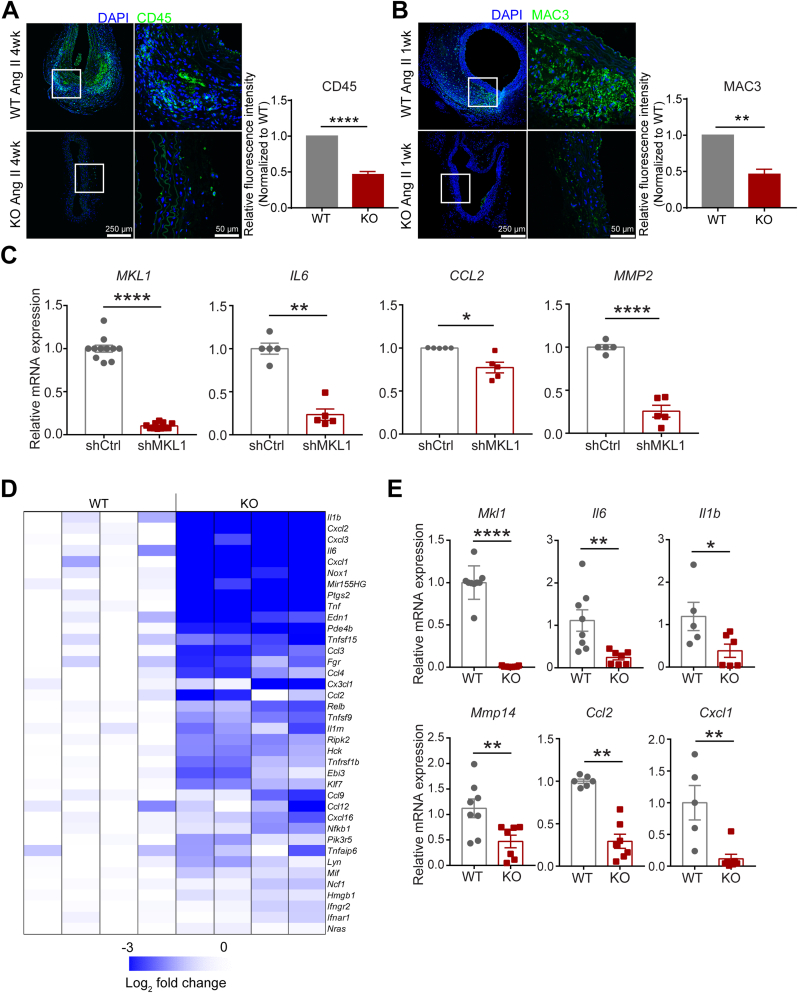
Depletion of MKL1 suppresses vascular inflammation. A and B, Representative confocal microscopy images for immunofluorescence staining and quantification (unpaired two-tailed Student's *t*-test) of the relative fluorescence intensity of the indicated proteins in cross-sections of the suprarenal aorta from *Mkl1*^*+/+*^*, Apoe*^*−/−*^ (WT) and *Mkl1*^*−/−*^*, Apoe*^*−/−*^ (KO) mice infused with Ang II for the indicated time (n = 3). C and E, qRT-PCR analysis of relative mRNA level of indicated inflammatory genes from HASMCs transduced with control or shMKL1 lentivirus for 72 h (n = 5, Mann–Whitney test for *MKL1* and *IL6,* Unpaired two-tailed Student's *t*-test for CCL2 and MMP2) (C) and primary cultured bone marrow derived macrophages (BMDMs) from WT versus KO mice infused with Ang II for 1 week (n = 5–7, Mann–Whitney test for *Mkl1* and *Cxcl1,* Unpaired two-tailed Student's *t*-test for *Il6*, *Il1b*, *Mmp14* and *Ccl2*) (E). D, Heatmap representation of transcriptomic profiling of key inflammatory mediators based on deep RNA-sequencing (RNA-seq) of BMDMs from KO versus WT mice infused with Ang II for 1 week. n = 4 per group. *P < 0.05, **P < 0.01, ****P < 0.0001.

Excessive activation of vascular inflammation has been documented as a critical determinant to AAA initiation and progression [2,7,40]. We asked if MKL1 deficiency could attenuate the activation of vascular inflammation and thus suppress AAA progression. Accordingly, we performed immunofluorescence staining for CD45 and MAC3 in suprarenal aortas. Our results showed that after 4 weeks of Ang II infusion, there were much less CD45 positive cells accumulated within the remodeled adventitia of suprarenal aortas in KOs compared with those from WT mice (Fig. 4A). Similarly, substantial accumulation of MAC3 positive macrophages in adventitia and medial SMC layer was only seen in suprarenal aortas from WT but not KO mice infused with Ang II for 1 week (Fig. 4B). These results are in agreement with previous findings [23,32,41], indicating a critical role for MKL1 in the activation of vascular inflammation triggered by Ang II infusion.

Given the importance of VSMCs and macrophages in governing vascular inflammation and AAA formation, and the induction of MKL1 in both cells types revealed by scRNA-seq in Fig. 1, we sought to determine if MKL1 can regulate proinflammatory gene expression in these two cell types. Depletion of MKL1 by lenti-shMKL1 in cultured HASMCs decreased mRNA levels of several important pathological markers of AAA, including *IL6*, *CCL2*, and *MMP2* [40,42,43] (Fig. 4C). This result was validated in 3 different HASMC isolates (data not shown) and MASMCs isolated from *Mkl1*^−/−^ versus *Mkl1*^*+/+*^ mice with and without the *Apoe*^*−/−*^ background (Supplementary Fig. 3A and B). On the other hand, since macrophages originating from bone marrow-derived monocytes contribute substantially to macrophage accumulation and subsequent transmural inflammation in AAA [44], we asked if MKL1 regulated inflammation in BMDMs. As such, we isolated bone marrow cells from WT versus KO mice and differentiated them for 1 week to macrophages followed by RNA isolation for bulk RNA-seq analysis and qRT-PCR validation. RNA-seq showed a widespread downregulation of mRNA levels of proinflammatory genes in BMDMs from KO mice compared with their counterparts from WT mice (Fig. 4D, E, GSE155298). Downregulation of several representative proinflammatory genes, including *Il6*, *Il1b*, *Mmp14*, *Ccl2*, and *Cxcl1* was validated by qRT-PCR. Together, the above results point to a critical role for MKL1 in activating a proinflammatory gene program in both VSMCs and macrophages.

### 3.5 MKL1 deficiency through gene deletion or inhibitor administration blocks Ang II-induced aortic dissection

**Fig. 5:**
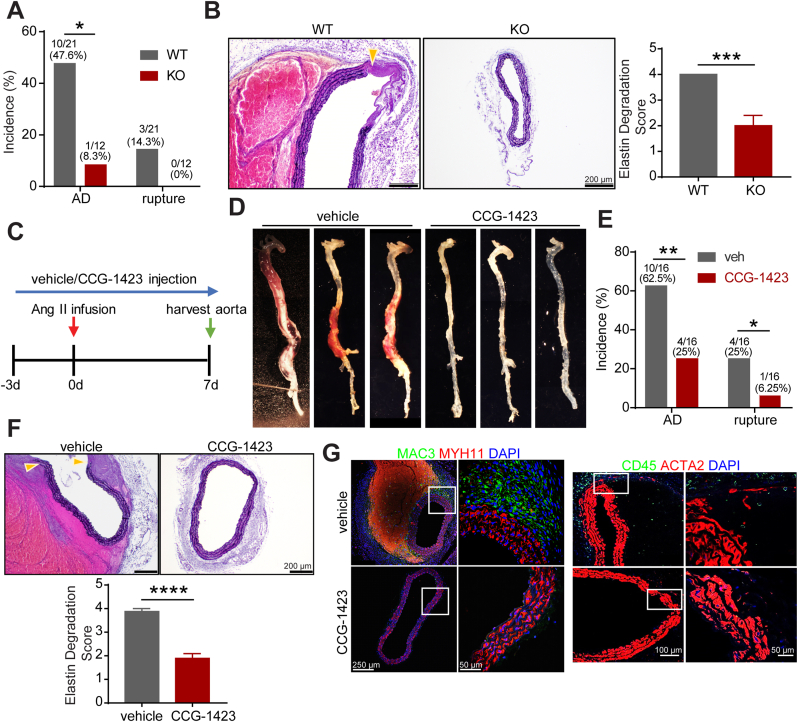
MKL1 deficiency through gene deletion or inhibitor administration prevents Ang II-induced aortic dissection. A, The incidence of aortic dissection (AD) and abdominal aortic rupture in *Mkl1*^*+/+*^*, Apoe*^*−/−*^ (WT) (n = 21) and *Mkl1*^*−/−*^*, Apoe*^*−/−*^ (KO) (n = 12) mice infused with Ang II for 1 week. Chi-square test. B, Representative images and quantification (unpaired two-tailed Student's *t*-test) of VVG staining of cross-sections of suprarenal aorta from WT and KO mice infused with Ang II for 1 week (n = 4). Location of extensive elastin degradation was labeled with yellow arrowhead. C, Timeline for vehicle or CCG-1423 (1 mg/kg/day) treatment. D, Representative en face photographs showing aortas from vehicle and CCG-1423 treated *Apoe*^*−/−*^ mice (n = 16). E, The incidence of AD and rupture in vehicle and CCG-1423 treated *Apoe*^*−/−*^ mice. Chi-square test. F, Representative images and the quantification (unpaired two-tailed Student's *t*-test) of VVG staining of cross-sections of suprarenal aorta from vehicle and CCG-1423 treated *Apoe*^*−/−*^ mice (n = 8). G, Representative confocal microscopy immunofluorescence staining of inflammatory mediators and SMC markers in cross-sections of suprarenal aorta from vehicle and CCG-1423 treated *Apoe*^*−/−*^ mice and the quantification (unpaired two-tailed Student's *t*-test) (n = 3). *P < 0.05, **P < 0.01, ***P < 0.001, ****P < 0.0001. (For interpretation of the references to color in this figure legend, the reader is referred to the Web version of this article.)

It has been documented that aortic dissection, originated from micro-ruptures in the intima and medial layer followed by the formation of intramural hematoma, is a common event preceding mature AAA formation in the Ang II-infused aneurysm model [32]. To determine if MKL1 deficiency can prevent this critical early event for AAA formation, we challenged WT and KO mice with Ang II for 1 week to induce aortic dissection. 1 week of Ang II infusion resulted in 47.6% (10/21) incidence of aortic dissection and 14.3% of aortic rupture in WT mice but only 14.3% (3/21) incidence of aortic dissection and no rupture in KOs (Fig. 5A). VVG staining of suprarenal aortas showed severe lamina elastin degradation and evident hematoma formation in WT aortas but such pathological features of aortic dissection were barely observed in KO mice (Fig. 5B). This result indicated that MKL1 is essential to the occurrence of aortic dissection. To determine whether MKL1 represents a potentially druggable target in aortic dissection and AAA, we treated *Apoe*^*−/−*^ mice with CCG-1423, a well-recognized pharmacological inhibitor of MKL1 [45]. Daily intraperitoneal injection of CCG-1423 starting 3 days before until 7 days after Ang II pump implantation (Fig. 5C) markedly suppressed aortic dissection (Fig. 5D). 1 week of Ang II infusion led to a 62.5% (10/16) incidence of aortic dissection in vehicle treated compared with only 25% (4/16) in CCG-1423 treated mice. During this interval, 4 out of 16 vehicle-treated and 1 of 16 CCG-1423 treated mice died due to aortic rupture (Fig. 5E). Similar to KO, CCG-1423 treated *Apoe*^*−/−*^ mice displayed relatively intact vasculature in suprarenal aortas compared with vehicle treated controls (Fig. 5F). Further, in accordance with our observation in KO mice, immunostaining for MAC3 and CD45 showed that the infiltration and accumulation of macrophages and leukocytes within the remodeled adventitia were largely abrogated by CCG-1423 administration (Fig. 5G). These results demonstrate that inhibition of MKL1, either by gene deletion or pharmacological inhibitor, blocked aortic dissection, a critical early event for AAA formation induced by Ang II infusion.

### 3.6 MKL1 deficiency inhibits p38MAPK activity in VSMCs

**Fig. 6:**
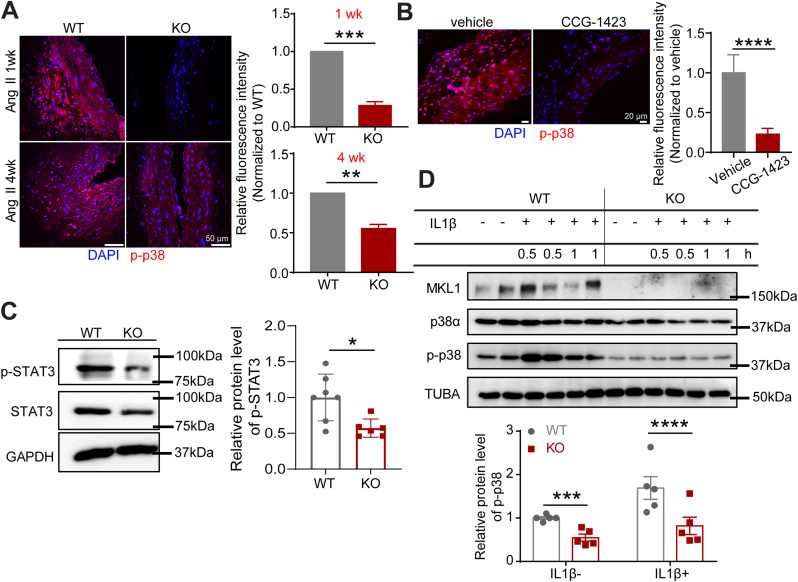
MKL1 deficiency inhibits p38MAPK activity in VSMCs. A and B, Representative confocal microscopy images for immunofluorescence staining and quantification (unpaired two-tailed Student's *t*-test) of the relative fluorescence intensity of p-p38 in cross-sections of suprarenal aortas from *Mkl1*^*+/+*^*, Apoe*^*−/−*^ (WT) and *Mkl1*^*−/−*^*, Apoe*^*−/−*^ (KO) mice infused with Ang II for the indicated time (A), or from vehicle and CCG-1423 treated *Apoe*^*−/−*^ mice infused with Ang II for 1 week (B) (n = 3/group). Quantifications were performed on at least 3 separate sections from 3 animals from each group, the average value from all the sections of a given animal was used for comparison. C, Representative Western blot images and quantification of indicated proteins in aorta homogenates of WT and KO mice infused with Ang II for 1 week (n = 7). Unpaired two-tailed Student's *t*-test. D, Representative Western blot images for the indicated proteins of mouse aortic SMCs (MASMCs) isolated from WT or KO mice treated with vehicle (IL1β-) or IL1β (5 ng/ml) for the indicated time, and the quantitation for the groups treated with vehicle and IL1β for 1 h (n = 5). Pair matched two-way ANOVA followed by Sidak multiple comparison test (D). Expression levels of p-STAT3 (C) and p-p38 (D) are normalized with GAPDH and total p38α, respectively. *P < 0.05, **P < 0.01, ***P < 0.001, ****P < 0.0001, NS: not significant.

The p38MAPK pathway is a pivotal activator of proinflammatory gene program [19]. In addition, p38MAPK was reported to mediate Ang II-induced phenotypic switching of VSMCs [20,30] and induce senescence contributing to aging associated diseases [46,47]. To gain insight into the mechanism underlying the protection from AAA exerted by MKL1 deficiency in mice, we examined the activation status of p38MAPK pathway in AAs after Ang II infusion via immunofluorescence staining for phosphorylated form of p38 (p-p38). The intensity of p-p38 staining was markedly reduced in suprarenal aortas from KO at both 1 and 4 weeks after Ang II infusion, as well as CCG-1423 treated *Apoe*^*−/−*^ mice with 1 week Ang II infusion relative to their individual control mice (Fig. 6A and B). It has been reported that p38MAPK can activate inflammation through STAT3 [48]. Western blot showed that levels of phosphorylated form of STAT3 (p-STAT3) were increased after 7 days Ang II infusion, indicating that the activation of STAT3 pathway may participate in Ang II-induced AAA formation (Supplementary Fig. 4A). Consistently, p-STAT3 was lower in aortas of KO mice with Ang II infusion for 1 week (Fig. 6C). Because IL1β is upregulated in human AAA and involved in Ang II-induced AAA formation [49,50], we used IL1β as a proinflammatory stimulus for in vitro cultured VSMCs. Western blot showed a reduction of p-p38 in MASMCs dispersed from KO mice compared with the counterparts from WT at both baseline levels and upon IL1β stimulation for 1 h (Fig. 6D). This result was reproduced in HASMCs treated with lenti-shMKL1 (Supplementary Fig. 4B and C). Taken together, these data support VSMC-MKL1 in the activation of p38MAPK, a key pathway governing both inflammation and senescence, ultimately contributing to AAA development.

### 3.7 MAPK14 (p38α) deficiency in SMCs ameliorates AAA formation and vascular senescence and inflammation

**Fig. 7:**
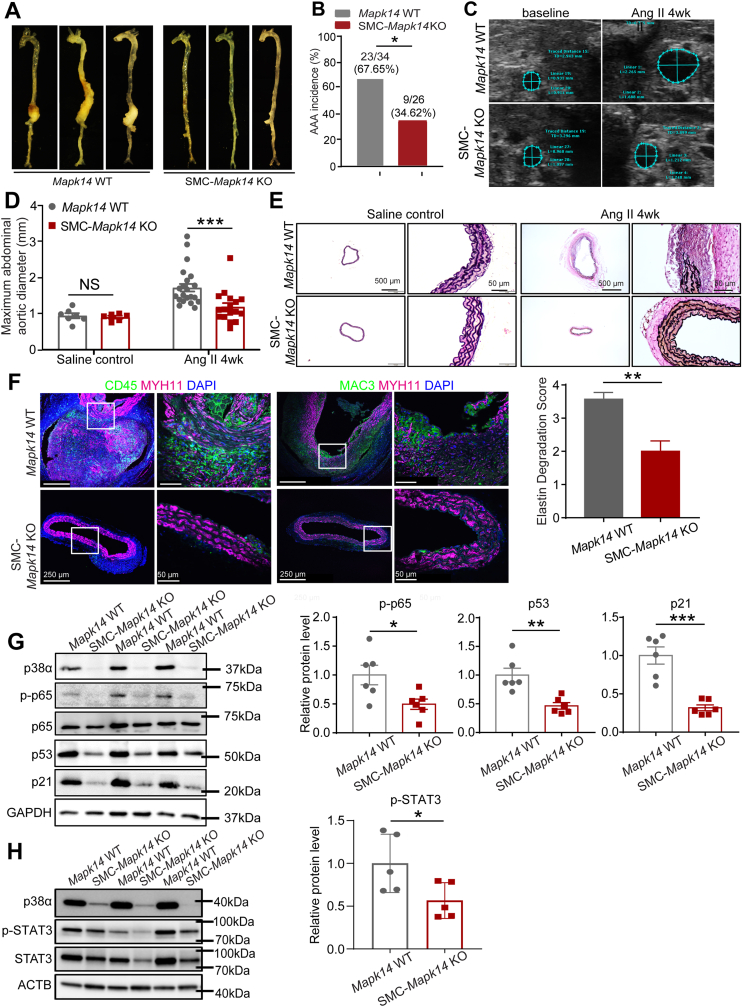
MAPK14 deficiency in VSMCs ameliorates AAA formation and vascular senescence and inflammation. A-F, All mice were infused with saline or Ang II for 4 weeks. A, Representative en face photographs showing aortas from *Mapk1*4 WT (*Sm22-Cre, Mapk14*^*+/+*^*, Apoe*^*−/−*^) and SMC*-Mapk14* KO (*Sm22-Cre, Mapk14*^*f/f*^*, Apoe*^*−/−*^) mice. B, The incidence of AAA in Ang II-infused *Mapk1*4 WT (n = 34, including n = 22 of *Mapk14*^*f/f*^*, Apoe*^*−/−*^ and n = 12 of *Sm22-Cre, Mapk14*^*+/+*^*, Apoe*^*−/−*^) and SMC*-Mapk14* KO (n = 26) mice. Chi-square test. C, Representative Doppler ultrasound images of abdominal aortas before (baseline) and 4 weeks after Ang II (Ang II 4 wk) (n = 10). D, The maximal abdominal aortic diameter (the width of the maximal expanded portion of suprarenal aorta) quantified from excised aortas as shown in A (n = 7 for *Mapk14*^*f/f*^*, Apoe*^*−/−*^ and n = 6 for SMC*-Mapk14* KO were included for saline control group; n = 24 for *Mapk14* WT and n = 17 for SMC*-Mapk14* KO were included for Ang II 4wk group). Two-way ANOVA followed by Sidak multiple comparison test. E, Representative images and quantification of elastin degradation (Unpaired two-tailed Student's *t*-test) from VVG staining of cross-sections of suprarenal aortas (n = 5). Quantifications were performed on at least 3 separate sections from 4 animals from each group, the average value from all the sections of a given animal was used for comparison. F, Representative confocal microscopy images for immunofluorescence staining of indicated proteins in cross-sections of the suprarenal aortas (n = 4). G and H, Western blot images and quantification of the indicated proteins in aorta homogenates from *Mapk1*4 WT and SMC*-Mapk14* KO mice infused with Ang II for 1 week (n = 6, G; n = 5, H). Unpaired two-tailed Student's *t*-test. *P < 0.05, **P < 0.01, ***P < 0.001, NS: no significant.

The aforementioned data suggest that activated p38MAPK pathway in VSMCs may cooperate with MKL1 to promote AAA progression. To test this, we generated hyperlipidemic, SMC-specific *Mapk14* knockout mice (*Sm22Cre,* *Mapk14*^*f/f*^*, Apoe*^*−/−*^) and WT littermate controls (*Mapk14*^*f/f*^*, Apoe*^*−/−*^). These mice breed normally and exhibit no apparent differences under physiological conditions (data not shown). *Mapk14* deficiency in VSMCs was verified by immunofluorescence staining in AAs (Supplementary Fig. 5). To directly address the specific role of VSMC-derived p38α in AAA, we challenged *Sm22Cre,* *Mapk14*^*f/f*^*, Apoe*^*−/−*^ (SMC-*Mapk14* KO) and two different *Mapk14* WT control mice, *Mapk14*^*f/f*^*, Apoe*^*−/−*^ and *Sm22Cre,* *Mapk14*^*+/+*^*, Apoe*^*−/−*^ with Ang II infusion for 4 weeks. Notably, *Mapk14* depletion in SMCs led to a significant decrease in both the severity (Fig. 7A) and incidence (Fig. 7B) of AAA in comparison to *Mapk14* WT mice. Echocardiography further demonstrated the marked dilation of suprarenal AAs in *Mapk14* WT mice compared with the modest dilation in mutant mice (Fig. 7C). While there was no significant difference in maximal diameter of suprarenal AAs and vessel wall integrity between *Mapk14* WT and SMC-*Mapk14* KO mice at baseline, *Mapk14* WT AAs displayed significantly increased maximal diameters and severely degraded elastin lamina relative to KO mice after exposure to Ang II infusion for 4 weeks (Fig. 7D and E). As described above, the protection of MKL1 deficiency against AAA development was associated with the suppression of vascular senescence and inflammation. We henceforth examined the role of SMC-derived *Mapk14* in these processes. Immunostaining showed that 4-week Ang II treatment in *Mapk14* WT mice induced a prominent accumulation of CD45 positive leucocytes and MAC3 positive macrophages within the adventitia and medial layer of suprarenal AAs. By contrast, CD45 positive or MAC3 positive cells were barely observed in these regions of aortas from Ang II-infused SMC-*Mapk14* KO mice (Fig. 7F). Consistently, Western blot revealed significantly reduced levels of phosphorylated form of p65 (p-p65) (Fig. 7G) and p-STAT3 (Fig. 7H) in AAs from SMC-*Mapk14* KO mice relative to *Mapk14* WT control mice infused with Ang II for 1 week. These results indicate that reduced inflammatory response may underlie, at least partially, the protection of SMC-deficiency of *Mapk14* against AAA formation. We also found that, senescence marker proteins, including p53 and p21 were significantly reduced in aortas from SMC-*Mapk14* KO mice (Fig. 7G). Consistently, activation of p38MAPK pathway via forced expression of Ad-MKK6, an upstream kinase of p38MAPK, upregulated p21 protein expression (Supplemental Fig. 6A), which was blunted by siRNA-mediated *MAPK14* knockdown (Supplemental Fig. 6B) in a SMC cell line, SKLMS, suggesting that activation of MAPK14 promoted SMC senescence. These in vivo and in vitro data are in line with the protective phenotype observed in MKL1 null mice. They further demonstrate that similar to MKL1, the lack of MAPK14 in VSMCs reduced the susceptibility to AAA formation and severity in AAA pathology induced by Ang II, at least partially via modulation of vascular inflammation and senescence.

## 4 Discussion

The present study demonstrated that deficiency of MKL1 prevented aortic dissection and AAA formation. Several lines of mechanistic evidence were provided supporting the pro-aneurysmal fashion of MKL1 in Ang II-induced AAA model. We first showed that MKL1 expression was induced in murine and human TAA and AAA lesions. This induction was found to be detrimental given that both genetic ablation and pharmacological inhibition of MKL1 strongly protected mice from aortic dissection and AAA. The mode of MKL1 action on AAA initiation and progression is likely through induction of senescence and inflammation, involving its positive regulation on p38MAPK, at least in VSMCs. Our study, for the first time, not only linked MKL1 induction to human aortic aneurysm, but also uncovered a critical pro-aneurysm pathway composed of MKL1/p38MAPK in VSMCs. Given the strong effect of MKL1 deficiency on mitigation of aortic dissection and AAA, MKL1 could be considered a promising therapeutic target for aortic aneurysm.

MKL1, together with MKL2 and MYOCD, belongs to Myocardin-related transcription factors (MRTFs). Though commonality of these three family members has been established as a cofactor of SRF to transactivate CArG-dependent genes and SMC differentiation [13,51,52], they each exhibit distinct roles in the cardiovascular system. While genetic studies from knockout mouse models revealed that both MYOCD and MKL2 are indispensable for embryonic development and homeostasis of adult blood vessels owing to their vital role in VSMC differentiation [15,53,54], global deficiency of MKL1 has no obvious effect on these physiological processes [14]. In contrast, MKL1 has recently emerged as a critical driver for several vascular disorders, including injury-induced stenosis, atherosclerosis, and hypoxic pulmonary hypertension [17,55]. This functional distinction between MKL1 and MYOCD is likely attributable to their differential tissue expression profile and regulatory gene programs triggered by vascular pathophysiological stimuli. Compared with MYOCD, whose expression is tightly restricted in VSMCs as a molecular switch of VSMC differentiation [12], MKL1 is ubiquitously expressed in different cell lineages and modulates disparate gene programs. Therefore, it is much expected that diverse biological processes resulting in vascular maladaptation, such as migration [17,56], proliferation [57], fibrosis [58], and apoptosis [57] are governed by MKL1 in a context–dependent manner. Our study, through both genetic and pharmacological approaches, demonstrated a strong role for MKL1 in promoting aortic dissection and AAA, further underscoring its detrimental role in different vascular complications.

While this manuscript was in preparation, a descriptive paper entitled “MRTF-A promotes angiotensin II-induced inflammatory response and aortic dissection in mice” was published in *PLoS One.* [59]. The data reported here confirms and substantially extends the latter study's findings in several important ways. First, we translated our findings of MKL1 induction in murine AAA and TAA to humans. Second, we focused on AAA development, the most common type of aortic aneurysm using a well-established AngII-infused hyperlipidemic AAA mouse model. Finally, we illuminated a mechanistic pathway involving p38MAPK activation of vascular inflammation and senescence to facilitate AAA development. We also wish to point out that the aortic dissection model utilized previously has a very low incidence according to the original report [60]. Therefore, it is currently unclear how the latter study showed such a high frequency of aortic dissection in control mice without a hyperlipidemic challenge. Finally, the dose of CCG compound reported previously [59] was more than 300-fold higher relative to the dosed used here, suggesting likely toxicity/off target in mice.

Another distinguishing feature of our study is the positive role of MKL1 in vascular senescence. An increased SA-β-Gal activity and higher expression of senescence markers, such as p16, p21 and p53, were observed in AAs of WT mice compared with MKL1 KO mice. This indicates that MKL1 might be essential to senescence activation during AAA development. This in vivo finding was confirmed in cultured aortic SMCs wherein MKL1 deficiency alleviated Ang II-induced expression of p16 (CDKN2A), a definitive senescence marker, at both mRNA and protein levels. It is widely accepted that senescent cells exhibit a unique senescence-associated secretory phenotype (SASP), characterized by the release of multiple cytokines, chemokines, growth factors and proteases to fuel a proinflammatory and proteolytic toxic microenvironment. As such, SASP underlies the pathogenesis of a variety of human diseases, including Alzheimer's disease [61], reinopathy [62], type 2 diabetes mellitus [63], and atherosclerosis [64]. Though the definitive role of senescence has yet to be established in aortic aneurysm, both GWAS and genetic deletion of senescence associated genes in mice have implicated a destructive role in this significant human health problem [9,36,37,65]. Our finding that deficiency of MKL1 blunts vascular senescence accompanied by the marked mitigation of aneurysm occurrence and pathology, not only suggests a novel role for MKL1 in promoting vascular cell senescence, but also supports the detrimental impact of senescence on aortic aneurysm formation.

The mechanism through which MKL1 activates vascular senescence is currently unknown. MKL1 has been reported as an important regulator to epigenetically activate NOX family genes [66] and NOX gene activation has been documented to contribute to cellular senescence [67]. It will be of importance to determine if NOX family members are involved in MKL1-mediated VSMC senescence and thus AAA formation. Nevertheless, based on our observation of MKL1 deficiency on both senescence and inflammation, one might postulate that MKL1 could be a nodal regulator of both processes and thus bridge sustained VSMC inflammation and senescence during AAA progression. It should be noted that the majority of cells with high SA-β-Gal activity in our study were localized in adventitia and its adjacent medial SMC layer, presumably adventitial fibroblasts and VSMCs. However, unambiguous definition of the role of cellular senescence in AAA formation requires rigorous tools, such as p16-3MR transgenic mice and *p16*^*tdTom*^ lineage tracing reporter system [68,69]. This is particularly important given the lack of reliable p16 antibodies for immunostaining in vivo and the extensive heterogeneity of cell components in aneurysmal vasculature.

The regulation of MKL1 on both senescence and inflammation is intriguing. Although senescent cells can propagate inflammation in both cell autonomous and paracrine manners, the activation of inflammation exerted by MKL1 in VSMC and macrophages, two major cells for aneurysm pathogenesis, may also be subject to modulation by senescence-independent pathway(s). Indeed, previous in vitro studies from Xu's group have reported that MKL1 activates pro-inflammatory gene program in different vascular cells, both transcriptionally as a co-activator to p65, and epigenetically as a chromatin modifier to establish an active chromatin status in the regulatory regions of proinflammatory genes [70,71]. Though we could not extend MKL1/p65 pathway to our study in aneurysm context, given that our Western blot failed to reveal consistent differences in p-p65 expression in aortas from the WT versus KO infused with AngII for 7 days (data not shown), we did find that the activation of p38MAPK, another well-known proinflammatory signal pathway, was suppressed upon MKL1 deficiency. This finding therefore suggests a novel pathway utilized by MKL1 to direct VSMC inflammation during AAA development. However, the detailed mechanism underlying this regulatory axis remains to be defined. We were unable to detect the physical interaction between MKL1 and p38α in VSMCs under different conditions (data not shown), indicative of the indirect influence of MKL1 on p38 activity. One possibility is that most of these proinflammatory genes activated by MKL1 encode inflammatory mediators, such as CCL2, IL6, and MMP2, which can activate p38 pathway in either autocrine or paracrine pathways. Finally, given that MKL1 is a bona fide cofactor for SRF to converge signal from actin dynamic to gene transcription, it will be of importance to determine if this regulatory event is dependent upon SRF/CArG or whether MKL1 interacts with another transcription factor and its *cis* regulatory element.

Current efforts to understand MKL1-mediated gene regulation have been largely focused on cellular actin dynamics, which determines nucleocytoplasmic shuttling of MKL1 for gene transcription [72]. However, there should exist additional regulatory layers influencing MKL1 activity beyond nuclear translocation, such as the direct modulation of MKL1 expression. Results from our studies in murine and human aneurysm samples demonstrated an induction of MKL1 in aneurysmal tissues. As supported by both immunostaining and single cell RNA-seq analysis, this induction could be attributable to increased *Mkl1* gene expression in multiple cell types, including different SMC and macrophage clusters, monocytes, pericytes, and myofbroblasts. We suspect that this universal MKL1 induction in different cell types may collectively promote AAA progression, though the contribution of MKL1 from individual cell types to AAA pathogenesis requires utilization of cell-specific *Mkl1* knockout mouse models. The molecular mechanism underlying *Mkl1* mRNA upregulation during aneurysm development is unknown in our current study. *Mkl1* has been reported as a target of miR-1-mediated posttranscriptional regulation [17]. Whether *Mkl1* mRNA induction results from decreased miR-1 expression or involves novel transcriptional pathway (s) during AAA development awaits future investigation.

We previously reported that VSMC deficiency of MAPK14 (p38a), the primary isoform of p38 family in VSMCs, is essential for ligation injury-induced neointima formation via promoting VSMC phenotypic modulation and inflammation [20]. Despite that activated p38MAPK has been frequently linked to aortic aneurysm, the causative role of p38 pathway in this disease has yet to be explored. Our results showed that SMC-specific depletion of MAPK14 recapitulated the protective phenotype seen in MKL1 global KO mice, similarly evidenced by decreased proinflammatory response and senescence marker expression. It should be noted, beyond the documented proinflammatory role of p38MAPK, this pathway has emerged as a critical activator of cellular senescence and contributes to multiple aging associated diseases such as cancer and cognitive decline [47,73]. The reduced senescence marker protein expression and proinflammatory response seen in SMC-specific MAPK14 knockout mice in the Ang II induced AAA model are in agreement with these published functions. However, the detailed molecular mechanisms underlying p38MAPK governance of vascular inflammation and senescence warrants further investigation.

In conclusion, our data demonstrate a critical role for MKL1 in promoting AAA formation via enhancing vascular senescence and inflammation. The mode of MKL1 action on AAA formation involves MAPK14, whose deficiency in VSMCs recapitulates the phenotype seen in *Mkl1* KO. Given that genetic and pharmacological inhibition of MKL1 ameliorates AAA formation and the strong induction of MKL1 expression in human AAA tissues, MKL1 represents a promising druggable target for pharmacologic intervention in the prevention and/or treatment of AAA.

## Declaration of competing interest

None. Dr. LeMaire serves as a consultant for Terumo Aortic and Baxter Healthcare; serves as a principal investigator for clinical studies sponsored by Terumo Aortic and CytoSorbants; and serves as a co-investigator for clinical studies sponsored by W.L. Gore & Associates.

## References

[1] Pinard A., Jones G.T., Milewicz D.M. (2019). Genetics of thoracic and abdominal aortic diseases. Circ. Res..

[2] Sakalihasan N., Limet R., Defawe O.D. (2005). Abdominal aortic aneurysm. Lancet.

[3] Nordon I.M., Hinchliffe R.J., Loftus I.M., Thompson M.M. (2011). Pathophysiology and epidemiology of abdominal aortic aneurysms. Nat. Rev. Cardiol..

[4] Davis F.M., Daugherty A., Lu H.S. (2019). Updates of recent aortic aneurysm research. Arterioscler. Thromb. Vasc. Biol..

[5] (2018). Abdominal aortic aneurysms. Nat. Rev. Dis. Prim..

[6] Quintana R.A., Taylor W.R. (2019). Cellular mechanisms of aortic aneurysm formation. Circ. Res..

[7] Brangsch J., Reimann C., Kaufmann J.O., Adams L.C., Onthank D.C., Thöne-Reineke C. (2019). Concurrent molecular magnetic resonance imaging of inflammatory activity and extracellular matrix degradation for the prediction of aneurysm rupture. Circ. Cardiovasc. Imag..

[8] Huang X., Zhang H., Liang X., Hong Y., Mao M., Han Q. (2019). Adipose-derived mesenchymal stem cells isolated from patients with abdominal aortic aneurysm exhibit senescence phenomena. Oxid. Med. Cell Longev..

[9] Chen H.Z., Wang F., Gao P., Pei J.F., Liu Y., Xu T.T. (2016). Age-associated sirtuin 1 reduction in vascular smooth muscle links vascular senescence and inflammation to abdominal aortic aneurysm. Circ. Res..

[10] Wang D.Z., Li S., Hockemeyer D., Sutherland L., Wang Z., Schratt G. (2002). Potentiation of serum response factor activity by a family of myocardin-related transcription factors. Proc. Natl. Acad. Sci. U.S.A..

[11] Olson E.N., Nordheim A. (2010). Linking actin dynamics and gene transcription to drive cellular motile functions. Nat. Rev. Mol. Cell Biol..

[12] Chen J., Kitchen C.M., Streb J.W., Miano J.M. (2002). Myocardin: a component of a molecular switch for smooth muscle differentiation. J. Mol. Cell. Cardiol..

[13] Du K.L., Chen M., Li J., Lepore J.J., Mericko P., Parmacek M.S. (2004). Megakaryoblastic leukemia factor-1 transduces cytoskeletal signals and induces smooth muscle cell differentiation from undifferentiated embryonic stem cells. J. Biol. Chem..

[14] Li S., Chang S., Qi X., Richardson J.A., Olson E.N. (2006). Requirement of a myocardin-related transcription factor for development of mammary myoepithelial cells. Mol. Cell. Biol..

[15] Huang J., Wang T., Wright A.C., Yang J., Zhou S., Li L. (2015). Myocardin is required for maintenance of vascular and visceral smooth muscle homeostasis during postnatal development. Proc. Natl. Acad. Sci. U.S.A..

[16] Hinohara K., Nakajima T., Yasunami M., Houda S., Sasaoka T., Yamamoto K. (2009). Megakaryoblastic leukemia factor-1 gene in the susceptibility to coronary artery disease. Hum. Genet..

[17] Minami T., Kuwahara K., Nakagawa Y., Takaoka M., Kinoshita H., Nakao K. (2012). Reciprocal expression of MRTF-A and myocardin is crucial for pathological vascular remodelling in mice. EMBO J..

[18] Osmanagic-Myers S., Kiss A., Manakanatas C., Hamza O., Sedlmayer F., Szabo P.L. (2019). Endothelial progerin expression causes cardiovascular pathology through an impaired mechanoresponse. J. Clin. Investig..

[19] Gupta J., Nebreda A.R. (2015). Roles of p38α mitogen-activated protein kinase in mouse models of inflammatory diseases and cancer. FEBS J..

[20] Wu W., Zhang W., Choi M., Zhao J., Gao P., Xue M. (2019). Vascular smooth muscle-MAPK14 is required for neointimal hyperplasia by suppressing VSMC differentiation and inducing proliferation and inflammation. Redox Biol..

[21] Nishida K., Yamaguchi O., Hirotani S., Hikoso S., Higuchi Y., Watanabe T. (2004). p38alpha mitogen-activated protein kinase plays a critical role in cardiomyocyte survival but not in cardiac hypertrophic growth in response to pressure overload. Mol. Cell. Biol..

[22] Miano J.M., Ramanan N., Georger M.A., de Mesy Bentley K.L., Emerson R.L., Balza R.O. (2004). Restricted inactivation of serum response factor to the cardiovascular system. Proc. Natl. Acad. Sci. U.S.A..

[23] Daugherty A., Manning M.W., Cassis L.A. (2000). Angiotensin II promotes atherosclerotic lesions and aneurysms in apolipoprotein E-deficient mice. J. Clin. Investig..

[24] Sharma N., Dev R., Belenchia A.M., Aroor A.R., Whaley-Connell A., Pulakat L. (2019). Deficiency of IL12p40 (interleukin 12 p40) promotes Ang II (angiotensin II)-Induced abdominal aortic aneurysm. Arterioscler. Thromb. Vasc. Biol..

[25] Martin-McNulty B., Vincelette J., Vergona R., Sullivan M.E., Wang Y.X. (2005). Noninvasive measurement of abdominal aortic aneurysms in intact mice by a high-frequency ultrasound imaging system. Ultrasound Med. Biol..

[26] Pelisek J., Hegenloh R., Bauer S., Metschl S., Pauli J., Glukha N. (2019). Biobanking: objectives, requirements, and future challenges-experiences from the Munich vascular Biobank. J. Clin. Med..

[27] Luo W., Wang Y., Zhang L., Ren P., Zhang C., Li Y. (2020). Critical role of cytosolic DNA and its sensing adaptor STING in aortic degeneration, dissection, and rupture. Circulation.

[28] Satoh K., Nigro P., Matoba T., O'Dell M.R., Cui Z., Shi X. (2009). Cyclophilin A enhances vascular oxidative stress and the development of angiotensin II-induced aortic aneurysms. Nat. Med..

[29] Varney S.D., Betts C.B., Zheng R., Wu L., Hinz B., Zhou J. (2016). Hic-5 is required for myofibroblast differentiation by regulating mechanically dependent MRTF-A nuclear accumulation. J. Cell Sci..

[30] Long X., Cowan S.L., Miano J.M. (2013). Mitogen-activated protein kinase 14 is a novel negative regulatory switch for the vascular smooth muscle cell contractile gene program. Arterioscler. Thromb. Vasc. Biol..

[31] Choi M., Lu Y.W., Zhao J., Wu M., Zhang W., Long X. (2020). Transcriptional control of a novel long noncoding RNA Mymsl in smooth muscle cells by a single Cis-element and its initial functional characterization in vessels. J. Mol. Cell. Cardiol..

[32] Saraff K., Babamusta F., Cassis L.A., Daugherty A. (2003). Aortic dissection precedes formation of aneurysms and atherosclerosis in angiotensin II-infused, apolipoprotein E-deficient mice. Arterioscler. Thromb. Vasc. Biol..

[33] Yamanouchi D., Morgan S., Kato K., Lengfeld J., Zhang F., Liu B. (2010). Effects of caspase inhibitor on angiotensin II-induced abdominal aortic aneurysm in apolipoprotein E-deficient mice. Arterioscler. Thromb. Vasc. Biol..

[34] Wu D., Ren P., Zheng Y., Zhang L., Xu G., Xie W. (2017). NLRP3 (nucleotide oligomerization domain-like receptor family, pyrin domain containing 3)-caspase-1 inflammasome degrades contractile proteins: implications for aortic biomechanical dysfunction and aneurysm and dissection formation. Arterioscler. Thromb. Vasc. Biol..

[35] Daugherty A., Manning M.W., Cassis L.A. (2001). Antagonism of AT2 receptors augments angiotensin II-induced abdominal aortic aneurysms and atherosclerosis. Br. J. Pharmacol..

[36] Liao S., Curci J.A., Kelley B.J., Sicard G.A., Thompson R.W. (2000). Accelerated replicative senescence of medial smooth muscle cells derived from abdominal aortic aneurysms compared to the adjacent inferior mesenteric artery. J. Surg. Res..

[37] Jeck W.R., Siebold A.P., Sharpless N.E. (2012). Review: a meta-analysis of GWAS and age-associated diseases. Aging Cell.

[38] Chen P.Y., Qin L., Li G., Malagon-Lopez J., Wang Z., Bergaya S. (2020). Smooth muscle cell reprogramming in aortic aneurysms. Cell stem cell.

[39] Clément M., Chappell J., Raffort J., Lareyre F., Vandestienne M., Taylor A.L. (2019). Vascular smooth muscle cell plasticity and autophagy in dissecting aortic aneurysms. Arterioscler. Thromb. Vasc. Biol..

[40] Harrison S.C., Smith A.J., Jones G.T., Swerdlow D.I., Rampuri R., Bown M.J. (2013). Interleukin-6 receptor pathways in abdominal aortic aneurysm. Eur. Heart J..

[41] Wang Y., Ait-Oufella H., Herbin O., Bonnin P., Ramkhelawon B., Taleb S. (2010). TGF-beta activity protects against inflammatory aortic aneurysm progression and complications in angiotensin II-infused mice. J. Clin. Investig..

[42] Longo G.M., Xiong W., Greiner T.C., Zhao Y., Fiotti N., Baxter B.T. (2002). Matrix metalloproteinases 2 and 9 work in concert to produce aortic aneurysms. J. Clin. Investig..

[43] Wang S., Zhang C., Zhang M., Liang B., Zhu H., Lee J. (2012). Activation of AMP-activated protein kinase α2 by nicotine instigates formation of abdominal aortic aneurysms in mice in vivo. Nat. Med..

[44] Raffort J., Lareyre F., Clement M., Hassen-Khodja R., Chinetti G., Mallat Z. (2017). Monocytes and macrophages in abdominal aortic aneurysm. Nat. Rev. Cardiol..

[45] Lundquist M.R., Storaska A.J., Liu T.C., Larsen S.D., Evans T., Neubig R.R. (2014). Redox modification of nuclear actin by MICAL-2 regulates SRF signaling. Cell.

[46] Freund A., Patil C.K., Campisi J. (2011). p38MAPK is a novel DNA damage response-independent regulator of the senescence-associated secretory phenotype. EMBO J..

[47] Brichkina A., Bertero T., Loh H.M., Nguyen N.T., Emelyanov A., Rigade S. (2016). p38MAPK builds a hyaluronan cancer niche to drive lung tumorigenesis. Gene Dev..

[48] Meng A., Zhang X., Shi Y. (2014). Role of p38 MAPK and STAT3 in lipopolysaccharide-stimulated mouse alveolar macrophages. Exp. Ther. Med..

[49] Juvonen J., Surcel H.M., Satta J., Teppo A.M., Bloigu A., Syrjälä H. (1997). Elevated circulating levels of inflammatory cytokines in patients with abdominal aortic aneurysm. Arterioscler. Thromb. Vasc. Biol..

[50] Vandestienne M., Zhang Y., Santos-Zas I., Al-Rifai R., Joffre J., Giraud A. (2021). TREM-1 orchestrates angiotensin II-induced monocyte trafficking and promotes experimental abdominal aortic aneurysm. J. Clin. Investig..

[51] Hinson J.S., Medlin M.D., Lockman K., Taylor J.M., Mack C.P. (2007). Smooth muscle cell-specific transcription is regulated by nuclear localization of the myocardin-related transcription factors. Am. J. Physiol. Heart Circ. Physiol..

[52] Wang Z., Wang D.Z., Hockemeyer D., McAnally J., Nordheim A., Olson E.N. (2004). Myocardin and ternary complex factors compete for SRF to control smooth muscle gene expression. Nature.

[53] Oh J., Richardson J.A., Olson E.N. (2005). Requirement of myocardin-related transcription factor-B for remodeling of branchial arch arteries and smooth muscle differentiation. Proc. Natl. Acad. Sci. U.S.A..

[54] Li S., Wang D.Z., Wang Z., Richardson J.A., Olson E.N. (2003). The serum response factor coactivator myocardin is required for vascular smooth muscle development. Proc. Natl. Acad. Sci. U.S.A..

[55] Chen D., Yang Y., Cheng X., Fang F., Xu G., Yuan Z. (2015). Megakaryocytic leukemia 1 directs a histone H3 lysine 4 methyltransferase complex to regulate hypoxic pulmonary hypertension. Hypertension.

[56] Sprenkeler E.G.G., Henriet S.S.V., Tool A.T.J., Kreft I.C., van der Bijl I., Aarts C.E.M. (2020). MKL1 deficiency results in a severe neutrophil motility defect due to impaired actin polymerization. Blood.

[57] An J., Naruse T.K., Hinohara K., Soejima Y., Sawabe M., Nakagawa Y. (2019). MRTF-A regulates proliferation and survival properties of pro-atherogenic macrophages. J. Mol. Cell. Cardiol..

[58] Behrmann A., Zhong D., Li L., Cheng S.L., Mead M., Ramachandran B. (2020). PTH/PTHrP receptor signaling restricts arterial fibrosis in diabetic LDLR(-/-) mice by inhibiting myocardin-related transcription factor relays. Circ. Res..

[59] Ito S., Hashimoto Y., Majima R., Nakao E., Aoki H., Nishihara M. (2020). MRTF-A promotes angiotensin II-induced inflammatory response and aortic dissection in mice. PloS One.

[60] Martín-Alonso M., García-Redondo A.B., Guo D., Camafeita E., Martínez F., Alfranca A. (2015). Deficiency of MMP17/MT4-MMP proteolytic activity predisposes to aortic aneurysm in mice. Circ. Res..

[61] Zhu Y., Armstrong J.L., Tchkonia T., Kirkland J.L. (2014). Cellular senescence and the senescent secretory phenotype in age-related chronic diseases. Curr. Opin. Clin. Nutr. Metab. Care.

[62] Oubaha M., Miloudi K., Dejda A., Guber V., Mawambo G., Germain M.A. (2016). Senescence-associated secretory phenotype contributes to pathological angiogenesis in retinopathy. Sci. Transl. Med..

[63] Khosla S., Farr J.N., Tchkonia T., Kirkland J.L. (2020). The role of cellular senescence in ageing and endocrine disease. Nat. Rev. Endocrinol..

[64] Minamino T., Komuro I. (2007). Vascular cell senescence: contribution to atherosclerosis. Circ. Res..

[65] Zhang W.M., Liu Y., Li T.T., Piao C.M., Liu O., Liu J.L. (2016). Sustained activation of ADP/P2ry12 signaling induces SMC senescence contributing to thoracic aortic aneurysm/dissection. J. Mol. Cell. Cardiol..

[66] Yu L., Yang G., Zhang X., Wang P., Weng X., Yang Y. (2018). Megakaryocytic leukemia 1 bridges epigenetic activation of NADPH oxidase in macrophages to cardiac ischemia-reperfusion injury. Circulation.

[67] Pagano P.J., Cifuentes-Pagano E. (2021). The enigmatic vascular NOX: from artifact to double agent of change: arthur C. Corcoran memorial lecture - 2019. Hypertension.

[68] Liu J.Y., Souroullas G.P., Diekman B.O., Krishnamurthy J., Hall B.M., Sorrentino J.A. (2019). Cells exhibiting strong p16 (INK4a) promoter activation in vivo display features of senescence. Proc. Natl. Acad. Sci. U.S.A..

[69] Jeon O.H., Kim C., Laberge R.M., Demaria M., Rathod S., Vasserot A.P. (2017). Local clearance of senescent cells attenuates the development of post-traumatic osteoarthritis and creates a pro-regenerative environment. Nat. Med..

[70] Yu L., Fang F., Dai X., Xu H., Qi X., Fang M. (2017). MKL1 defines the H3K4Me3 landscape for NF-κB dependent inflammatory response. Sci. Rep..

[71] Yu L., Weng X., Liang P., Dai X., Wu X., Xu H. (2014). MRTF-A mediates LPS-induced pro-inflammatory transcription by interacting with the COMPASS complex. J. Cell Sci..

[72] Miralles F., Posern G., Zaromytidou A.I., Treisman R. (2003). Actin dynamics control SRF activity by regulation of its coactivator MAL. Cell.

[73] Moreno-Cugnon L., Revuelta M., Arrizabalaga O., Colie S., Moreno-Valladares M., Jimenez-Blasco D. (2019). Neuronal p38α mediates age-associated neural stem cell exhaustion and cognitive decline. Aging Cell.

