# Supplementary material for: MKL1 cooperates with p38MAPK to promote vascular senescence, inflammation, and abdominal aortic aneurysm
Source: Redox Biol. 2021 Feb 20;41:101903. doi: 10.1016/j.redox.2021.101903 (PMC7937568; doi:10.1016/j.redox.2021.101903)
Supplement: Multimedia component 1 [file mmc1.docx]

**Supplementary Figures**

**
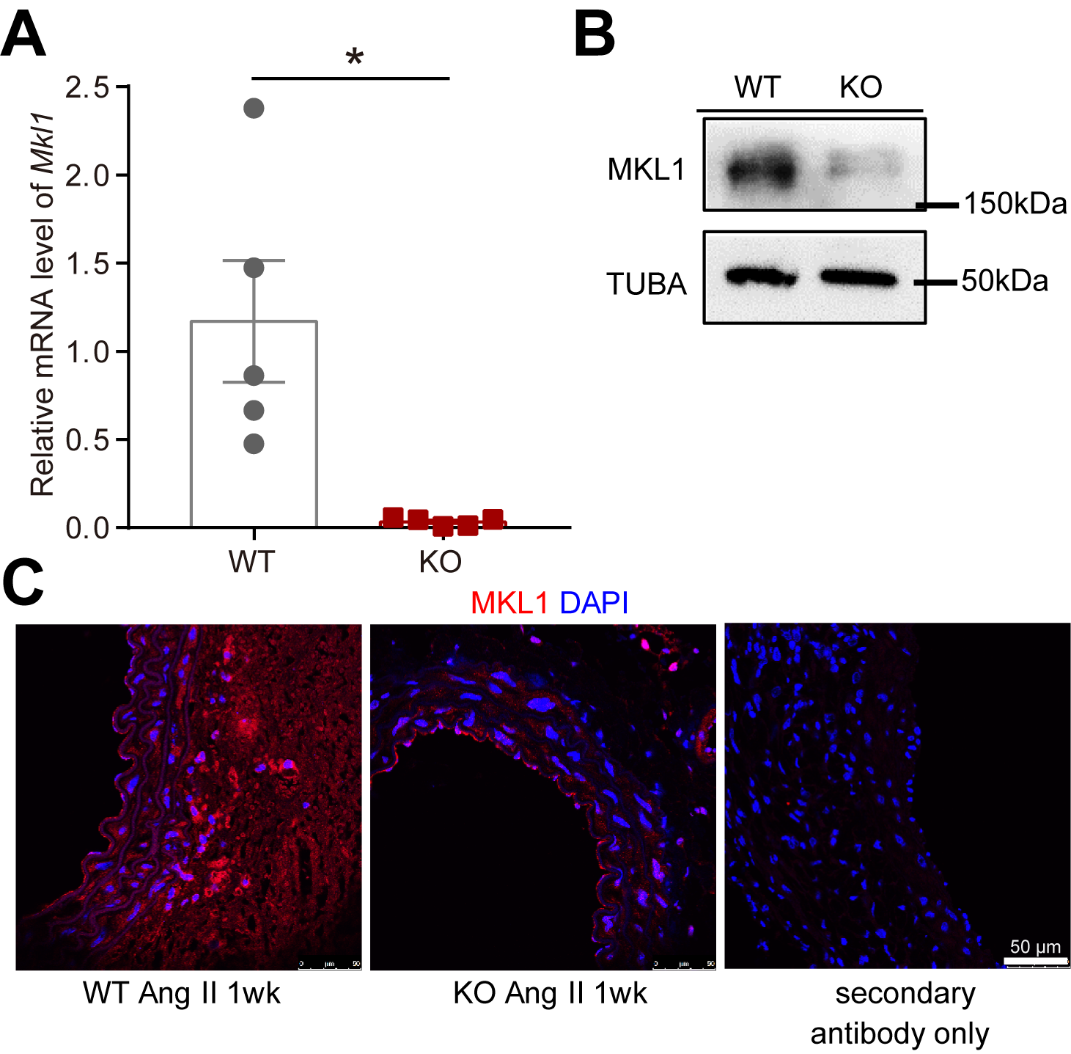
**

**Supplementary Figure 1.** **Validation of MKL1 deletion in aortas in MKL1 knockout mice. A** and **B,** Quantitative Real-Time Reverse Transcription PCR (qRT-PCR) analysis (n=5) (**A**) and Western blot images (n=7) (**B**) of MKL1 in aorta homogenates of WT and KO mice. **C,** Representative confocal microscopy immunofluorescence staining in cross-sections of the suprarenal aorta from mice infused with Ang II for 1 week. MKL1 antibody specificity was validated with KO and secondary antibody only controls (n=3). Unpaired two-tailed Student’s t test, *P<0.05.


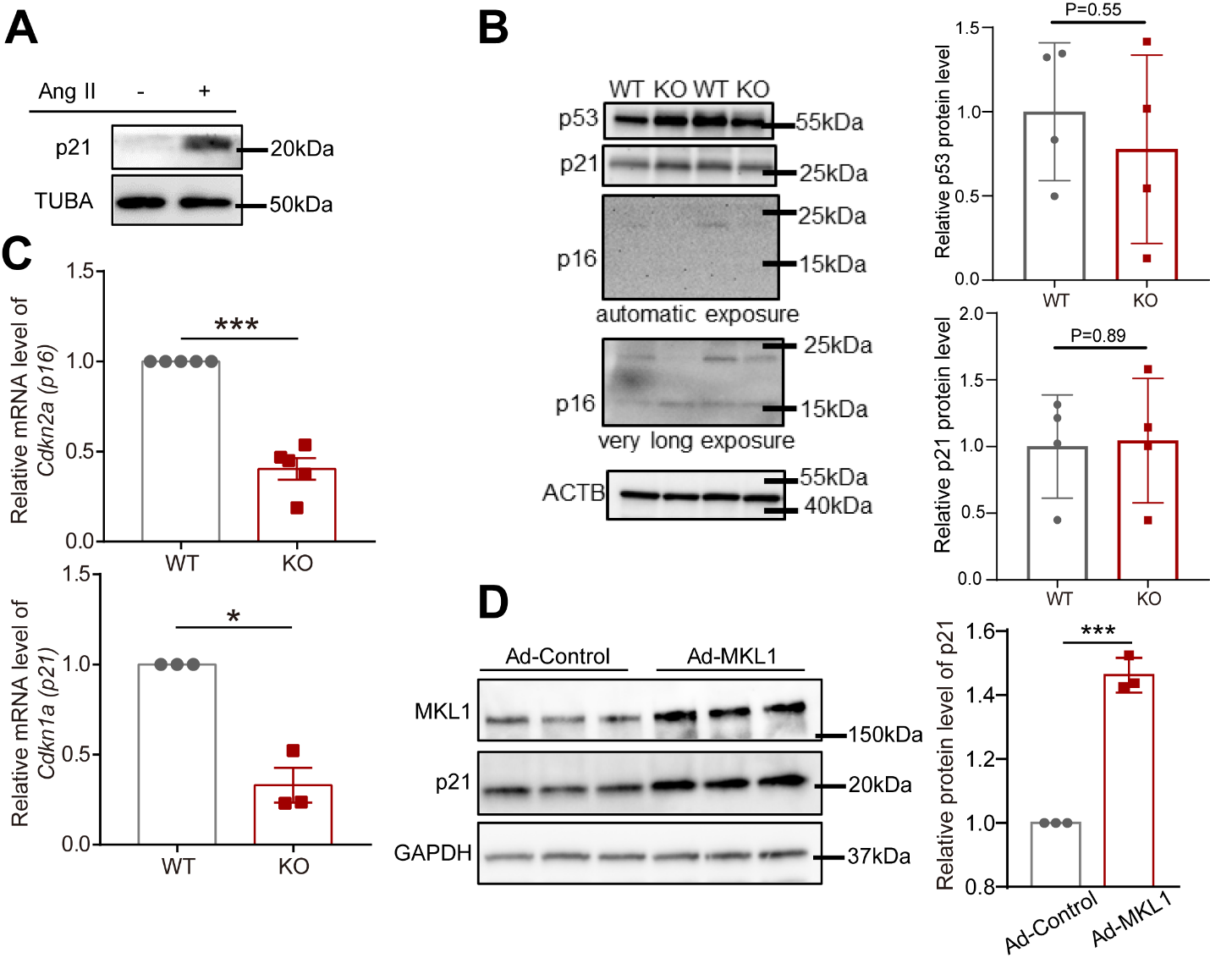


**Supplementary Figure 2.** **MKL1 is a positive regulator of vascular senescence. A,** Representative Western blot images of p21 in aorta homogenates of WT mice exposed to saline or Ang II for 1 week (n=6). **B,** Representative Western blot images of the indicated proteins in aorta homogenates of WT (*Mkl1^+/+^* *Apoe*^-/-^) and KO (*Mkl1^-/-^* *Apoe*^-/-^) mice infused with saline for 1 week and the quantitation (n=4). **C,** qRT-PCR analysis of relative levels of senescence markers from mouse aortic SMCs (MASMCs) isolated from *Mkl1^+/+^* (WT) *Mkl1^-/-^* (KO) mice treated with Ang II (10^-6^ M) or vehicle treatment for 24-48 h (n=5). **D,** Representative Western blot analysis of p21 in human aortic smooth muscle cells (HASMCs) transduced with control or Ad-MKL1 for 72 h (n=3). Unpaired two-tailed Student’s t test, *P<0.05, **P<0.01, ***P<0.001.


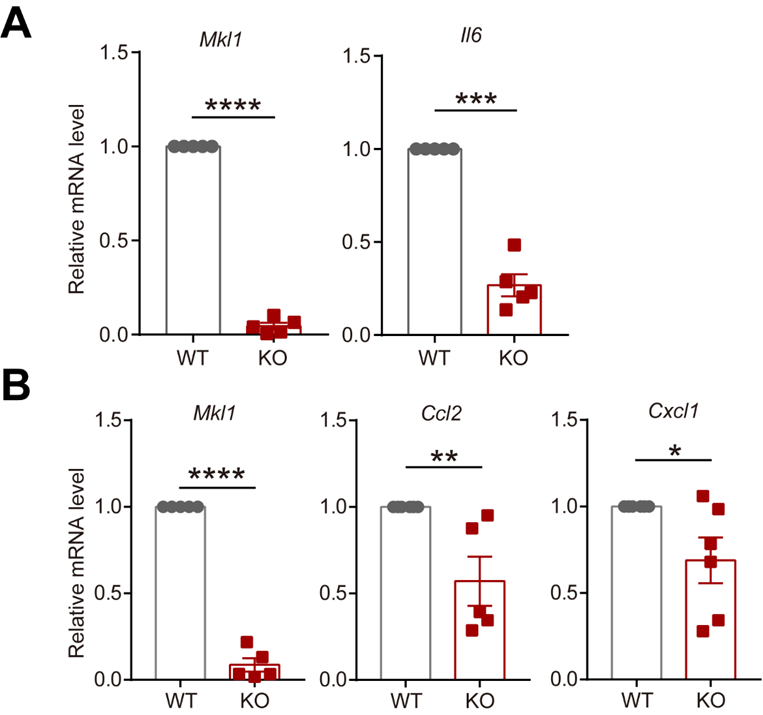


**Supplementary Figure 3.** **Depletion of MKL1 suppresses vascular inflammation. A** and **B,** qRT-PCR analysis of relative mRNA levels of indicated proinflammatory genes in MASMCs isolated from *Mkl1^+/+^* (WT) and *Mkl1^-/-^* (KO) mice (**A**) or *Mkl1^+/+^* *Apoe*^-/-^ (WT) and *Mkl1^-/-^* *Apoe*^-/-^ (KO) mice (**B**) (n>=5). Unpaired two-tailed Student’s t test, *P<0.05, **P<0.01, ***P<0.001, ****P<0.0001.


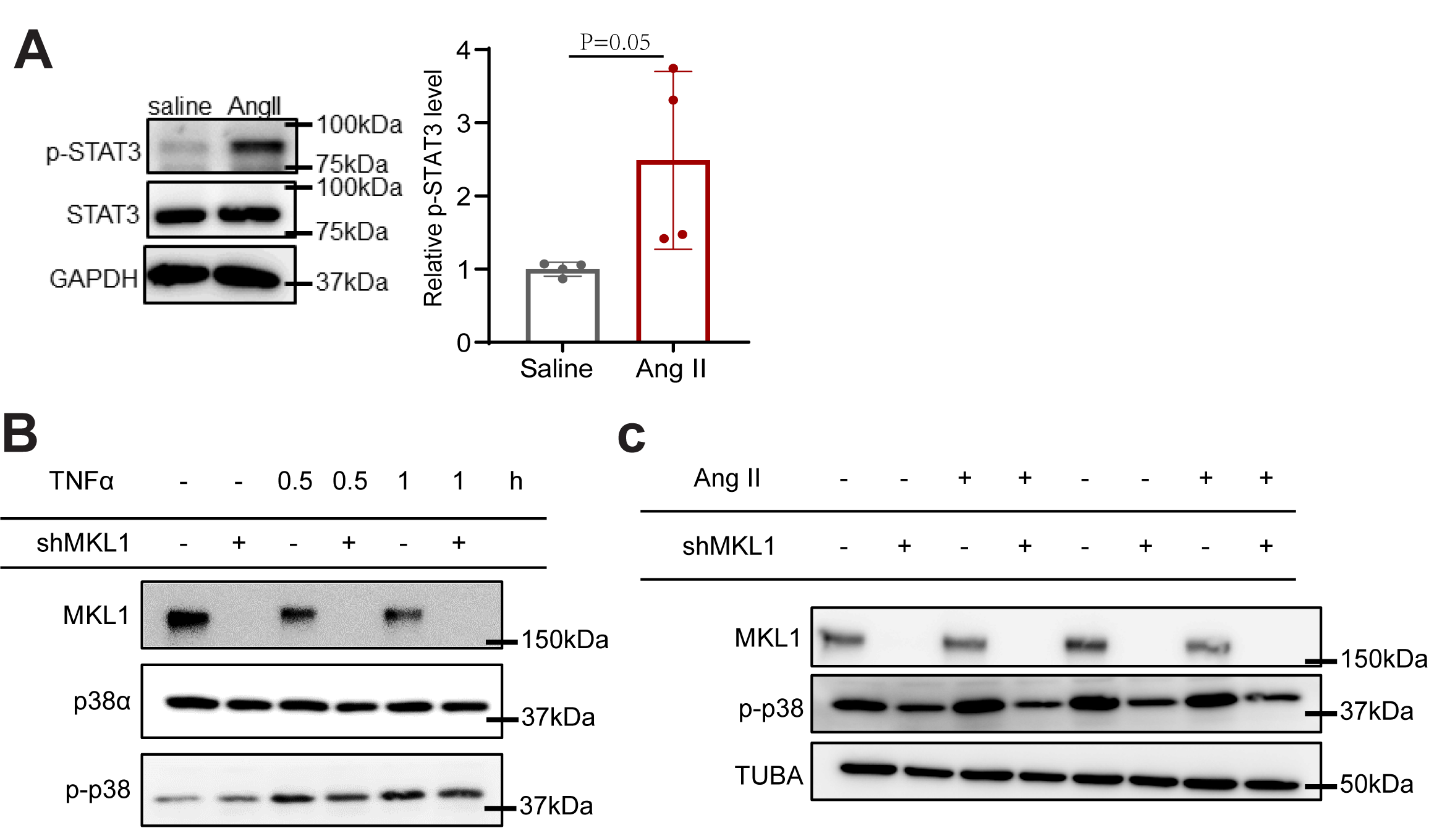


**Supplementary Figure 4. STAT3 pathways is activated in Ang II-induced AAA model and depletion of MKL1 in cultured VSMCs inhibits p38MAPK activity.** **A.** Representative Western blot images of the indicated proteins in aorta homogenates of *Apoe*^-/-^ mice infused with Ang II for 1 week and the quantitation (n=4). **B** and **C,** Representative Western blot images of the indicated proteins from HASMCs transduced with control or shMKL1 lentivirus for 72 h followed by TNFα (10 ng/ml) or vehicle treatment for the indicated time (**B**, n=6), or Ang II (10^-7^M) and vehicle treatment for 24 h (**C**, n=4).


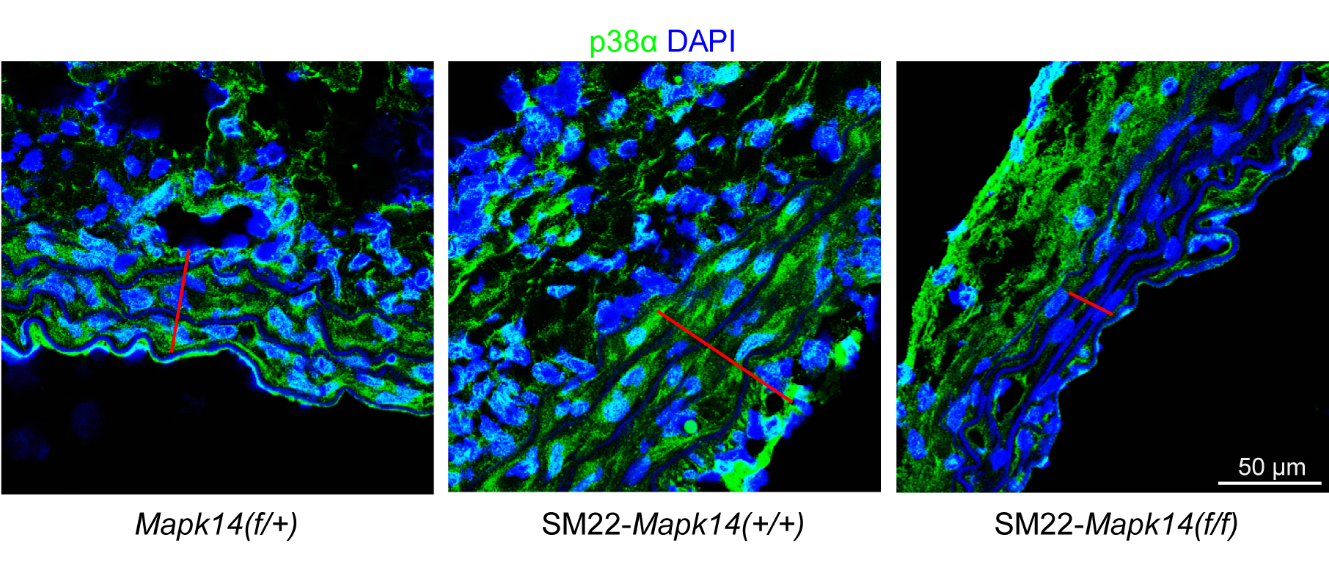


**Supplementary Figure 5. Validation of SMC-specific deletion of *Mapk14* in aortas in SMC-*Mapk14* KO mice.** Immunostaining for MAPK14 (p38α) in aortas from *Mapk14* WT control (*Mapk14* (f/+), SM22-*Mapk14* (+/+)), and SMC-*Mapk14* KO (SM22-*Mapk14* (f/f)) mice (n=3).

**
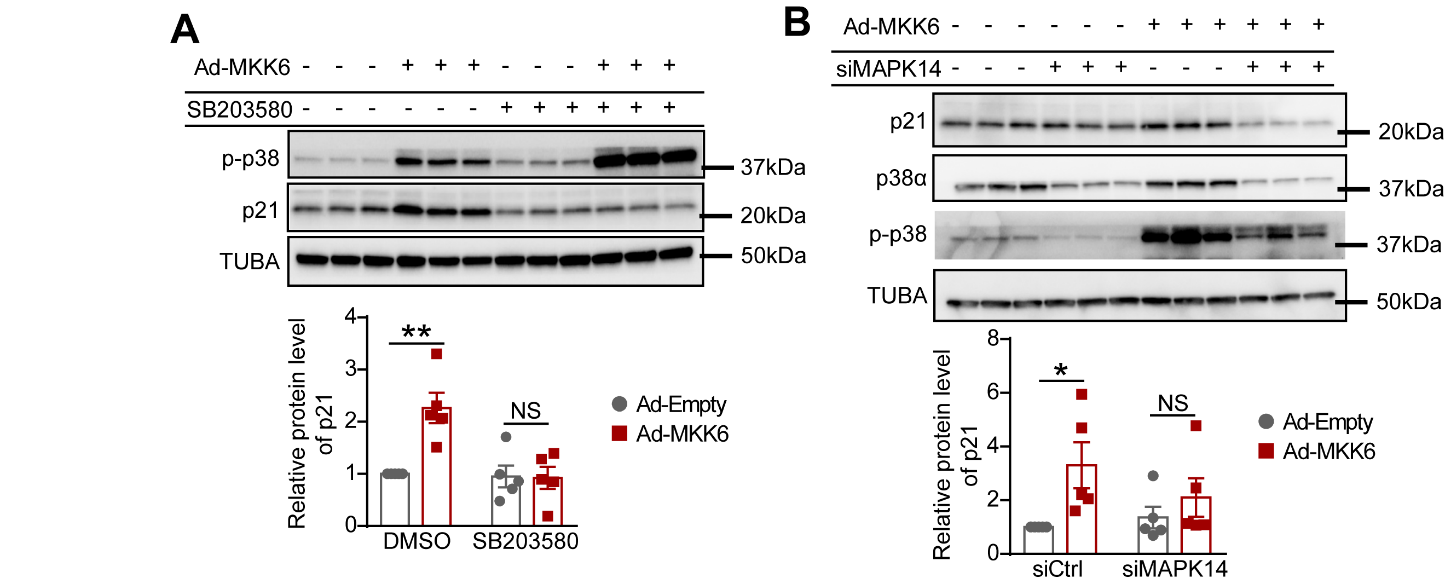
**

**Supplementary Figure 6. p38MAPK activation positively regulates p21 protein expression. A,** SKLMS±Ad-MKK6 (MOI=50) for 24 h followed by the incubation of SB203580 (10um) for 48 h prior to protein extraction, representative Western blot for the indicated proteins and the quantitation of p21 (n=5) were shown; **B,** SKLMS ± siMAPK14 for 24 h followed by the transduction of Ad-Empty or Ad-MKK6 (MOI=50) for 24 h prior to protein extraction, representative Western blot for the indicated proteins and quantitation of p21 were shown (n=5, 3 replicates/experiment);

**Supplementary Tables**

**Supplementary Table 1. Patient Characteristics of ATAA**

| **Characteristics** | **Control**  **(n=10)** | **ATAA**  **(n=16)** |
| --- | --- | --- |
| Age (y) | 64±7.4 | 63.4±10.2 |
| Men | 5 (50%) | 8 (50%) |
| Hypertension | 8 (80%) | 13 (81%) |
| COPD | 0 | 0 |
| History of smoking | 2 (20%) | 6 (38%) |
| Use of antilipid medication | 3 (30%) | 7 (44%) |
| Use of COX inhibitor | 2 (20%) | 4 (25%) |
| Aortic diameter (cm) | NA | 5.4±0.8 |

Data are expressed as a number (percent) or as the mean ± standard deviation. ATAA indicates ascending thoracic aortic tissue from patients with ascending thoracic aortic aneurysm; COPD, chronic obstructive pulmonary disease; COX, cyclooxygenase; NA, not available.

**Supplementary Table 2. Patient characteristics from Munich Vascular Biobank**

| **Characteristics** | **Controls**  **(n=6)** | **AAA**  **(n=24)** |
| --- | --- | --- |
| Diameter (cm) | NA | 5.8 ± 0.7 |
| Age (mean ± SEM) | 40 ± 2.8 | 72.5 ± 5.3 |
| Gender (male/female) | 5 m/1 fem | 20 m/4 fem |
| Hypertension (%) | 0% (0/6) | 92% (22/24) |
| Diabetes (%) | 0% (0/6) | 8% (2/24) |
| History of smoking (%) | 16% (1/6) | 88% (21/24) |

**Supplementary Table 3. Information of primary and secondary antibodies**

| **Primary Antibodies for Western Blot** | | | |
| --- | --- | --- | --- |
| **Target antigen** | **Vendor or Source** | **Catalog #** | **Working concentration** |
| MKL1 | Bethyl Laboratories | A302-201A | 1:1000 |
| MKL1 | Dr. Akiko Tabuchi (University of Toyama) | NA | 1:500 |
| p16^Ink4a^ | Abcam | ab108349 | 1:1000 |
| p16^Ink4a^ | Abcam | ab211542 | 1:1000 |
| p53 | Cell Signaling Technology | 2524 | 1:1000 |
| p21 | Millipore Sigma | 05-345 | 1:1000 |
| p38α | Santa Cruz Biotechnology | sc-535 | 1:500 |
| phospho-p38 | Cell Signaling Technology | 9211 | 1:1000 |
| GAPDH | Cell Signaling Technology | 5174 | 1:2000 |
| ACTB | Sigma-Aldrich | A5441 | 1:5000 |
| TUBA | Sigma-Aldrich | T5168 | 1:5000 |
| Phospho-MAPKAPK-2 | Cell Signaling Technology | 3007 | 1:1000 |
| phospho-p38 | Cell Signaling Technology | 4511 | 1:1000 |
| Phospho-P65 | Cell Signaling Technology | 3033 | 1:500 |
| NF-κB p65 | Cell Signaling Technology | 8242 | 1:1000 |
| Stat3α | Cell Signaling Technology | 8768 | 1:1000 |
| Phospho-Stat3 | Cell Signaling Technology | 9145 | 1:1000 |
| **Primary Antibodies for Immunofluorescence** | |  |  |
| **Target antigen** | **Vendor or Source** | **Catalog #** | **Working concentration** |
| MKL1^1^ | Dr. Akiko Tabuchi (University of Toyama) | NA | 1:50 |
| MYH11 | Alfa Aesar | BT-562 | 1:250 |
| CD45 | BD Pharmingen | 550566 | 1:50 |
| CD107b (MAC3) | BioLegend | 108512 | 1:50 |
| phospho-p38 | Cell Signaling Technology | 4511 | 1:100 |
| Actin, α-Smooth Muscle - Cy3 | Millipore Sigma | C6198 | 1:500 |

| **Secondary Antibodies** | | | |
| --- | --- | --- | --- |
| **Name** | **Vendor or Source** | **Catalog #** | **Working**  **concentration** |
| CF594A  Goat Anti-Guinea Pig IgG (H+L) | Biotium | 20118-1 | 1:1000 |
| Alexa Fluor 488  goat anti-mouse IgG (H+L) | Invitrogen/Thermo Fisher Scientific | A11001 | 1:500 |
| Alexa Fluor 488  goat anti-rabbit IgG (H+L) | Invitrogen/Thermo Fisher Scientific | A11034 | 1:500 |
| Alexa Fluor 488  goat anti-rat IgG (H+L) | Invitrogen/Thermo Fisher Scientific | A11006 | 1:500 |
| Alexa Fluor 555  goat anti-mouse IgG (H+L) | Invitrogen/Thermo Fisher Scientific | A21424 | 1:500 |
| Alexa Fluor 555  goat anti-rabbit IgG (H+L) | Invitrogen/Thermo Fisher Scientific | A27039 | 1:500 |
| Alexa Fluor 647  goat anti-rabbit IgG (H+L) | Invitrogen/Thermo Fisher Scientific | A21245 | 1:500 |
| Alexa Fluor 647  donkey anti-mouse IgG (H+L) | Invitrogen/Thermo Fisher Scientific | A31571 | 1:500 |
| Rabbit anti-Guinea Pig IgG (H+L), HRP | Invitrogen/Thermo Fisher Scientific | 614620 | 1:5000 |
| rabbit anti-Mouse IgG (H+L), HRP | Invitrogen/Thermo Fisher Scientific | 31450 | 1:5000 |
| Goat anti-Rabbit IgG (H+L), HRP | Invitrogen/Thermo Fisher Scientific | 31460 | 1:5000 |

**Supplementary Table 4. Sequence information of qRT-PCR primers**

| **Species** | **Gene name** | **Forward primer sequence** | **Reverse primer**  **sequence** |
| --- | --- | --- | --- |
| human | *18S* | ATGGGCGGCGGAAAATAGC | TCTTGGTGAGGTCAATGTCTGC |
| human | *MKL1* | CAGCCTGAAGGAAGCCATC | GCCCATCGGAAGTTGAGAC |
| human | *CDKN2A* | CCCAACGCACCGAATAGTTA | ACCAGCGTGTCCAGGAAG |
| human | *CDKN1A* | GCTATTTTGTCCTTGGGCTG | AAGTCGAAGTTCCATCGCTC |
| human | *IL6* | GTGTTGCCTGCTGCCTTC | AGTGCCTCTTTGCTGCTTTC |
| human | *CCL2* | CTGTGCCTGCTGCTCATAG | CTTGCTGCTGGTGATTCTTC |
| human | *MMP2* | CACAGCCAACTACGATGATG | AAGGTCAATGTCAGGAGAGG |
| mouse | *Hprt* | TGGCCCTCTGTGTGCTCAA | TGATCATTACAGTAGCTCTTCAGTCTGA |
| mouse | *Mkl1* | CCTGCTCCCACACTCATC | CCCCTTGTCCTGCTTCTG |
| mouse | *Cdkn2a* | AATCTCCGCGAGGAAAGC | GTCTGCAGCGGACTCCAT |
| mouse | *Cdkn1a* | CAGATCCACAGCGATATCCAG | AGAGACAACGGCACACTTTG |
| mouse | *Ccl2* | TCTCTCTTCCTCCACCAC | CTCTCCAGCCTACTCATTG |
| mouse | *Il6* | ACAAAGAAATGATGGATGCTACC | GTATCTCTCTGAAGGACTCTGG |
| mouse | *Mmp14* | TTCGTGTTGCCTGATGACGA | TTCCCGTCACAGATGTTGGG |
| mouse | *Cxcl1* | GTCATAGCCACACTCAAGAATG | GAACAAGCAGAACTGAACTACC |
| mouse | *Cxcl5* | TCAGTCATAGCCGCAACG | GGGTCAGAGTCCTCAGAAATC |
| mouse | *Myh11* | TGCCGACACAGCCTACAGAAG | GGACGCCACCACAGCCAAG |
| mouse | *Mmp2* | TGGAATGCCATCCCTGATAA | AGCCCAGCCAGTCTGATTTG |

**Supplementary Table 5. Blood cell count for WT versus MKL1 KO mice infused with Ang II for 4 weeks and the quantitation**

**Reference:**

1. Kaneda M, Sakagami H, Hida Y, Ohtsuka T, Satou N, Ishibashi Y, Fukuchi M, Krysiak A, Ishikawa M, Ihara D, Kalita K and Tabuchi A. Synaptic localisation of SRF coactivators, MKL1 and MKL2, and their role in dendritic spine morphology. *Sci Rep*. 2018;8:727.
